# Supplementary material for: Accelerated drug development using a digital formulator and a self-driving tableting data factory
Source: Nat Commun. 2026 Apr 1;17:4739. doi: 10.1038/s41467-026-71204-6 (PMC13216267; doi:10.1038/s41467-026-71204-6)
Supplement: Supplementary file 1 — Supplementary information [file 41467_2026_71204_MOESM1_ESM.pdf]

Supplementary Information for "Accelerated drug development using a digital formulator and a self-driving tableting data factory"

Faisal Abbas[1^] & Mohammad Salehian[1^], Peter Hou[1], Jonathan Moores[1], Jonathan Goldie[1], Alexandros Tsioutsios[1], Theo Tait[1], Victor Portela[2], Quentin Boulay[3], Roland Thiolliere[3], Ashley Stark[4], Jean-Jacques Schwartz[4], Jerome Guerin[4], Andrew G. P. Maloney[5], Alexandru A. Moldovan[5], Gavin K. Reynolds[6], Jérôme Mantanus[7], Catriona Clark[1], Paul Chapman[2], Alastair Florence[1], Daniel Markl[1*]

[^] Authors contributed equally.
[*] Corresponding author, Email: daniel.markl@strath.ac.uk

[1] CMAC, Strathclyde Institute of Pharmacy and Biomedical Science (SIPBS), University of Strathclyde, Glasgow, G1 1RD, UK
[2] Glasgow School of Art, Glasgow, G3 6RQ , UK
[3] Medelpharm, ZAC des Malettes, 615 Rue du Chat Botté, 01700 Beynost, France
[4] DEC Group, Chemin du Dévent 3, 1024 Ecublens, Switzerland
[5] The Cambridge Crystallographic Data Centre, 12 Union Road, Cambridge, CB2 1EZ, UK
[6] Sustainable Innovation & Transformational Excellence (xSITE), Pharmaceutical Technology & Development, Operations, AstraZeneca UK Limited, Macclesfield, SK10 2NA, UK
[7] UCB S.A, 60 Allée de la Recherche, 1070 Brussels, Belgium

Contents

1 Materials 4

2 Hybrid System of Models 6

2.1 Data Summary 6

2.2 Validation of FFC mixture model 10

2.3 Development and validation of process models 10

2.4 Model performance summary 13

2.5 Relative feature importance analysis 15

3 Tableting data factory 16

3.1 Make & test 16

3.1.1 List of instruments 16

3.1.2 Powder transportation unit (TU) 16

3.1.3 Robotic fingers 17

3.1.4 Automated tablet separator (ATS) 19

3.1.5 Customised cleaning unit (CU) for TU 19

3.1.6 Process analysis with near-infrared (NIR) spectroscopy 20

3.1.7 Orchestration system 20

3.1.8 Validation of tableting data factory 21

3.1.9 Assessment of powder loss on content uniformity 24

3.2 Experimental agent 26

3.2.1 Background of Bayesian optimisation 26

3.2.2 Digital integration of optimisation frameworks with tableting data factory 26

3.2.3 Multi-output Bayesian optimisation (MOBO) for rapid scale-up assessment 27

4 Demonstration of platform workflow 27

4.1 Formulations 27

4.2 Digital formulator 28

4.2.1 Flowability model performance 29

4.2.2 Parameterisation of distributions for API particle size assessment on blend and tablet properties 29
4.2.3 Sensitivity analysis of optimal formulations 33
4.3 Tableting data factory 38
4.3.1 Near-infrared (NIR) spectroscopy 38
4.3.2 Physics-informed Bayesian optimisation (PIBO) 41
4.3.3 Multi-output Bayesian optimisation (MOBO) for rapid scale-up assessment 44
4.3.4 Disintegration testing 46
5 Tablet manufacturing 47
5.1 Extended reality enabled process monitoring 47
5.2 Implementation of extended reality 48
6 Comparison to the state of the art 49
Supplementary references 51

1 Materials

Supplementary Table 1 provides a complete list of all materials used in this study, including the specific chemical grade and supplier information. Since many pharmaceutical compounds exist in multiple grades, we assigned a unique Material ID to each material. This notation is used consistently throughout the manuscript and Supplementary Information to avoid ambiguity when referring to materials with similar chemical names. This table ensures full traceability and reproducibility by allowing readers to identify the exact source and grade of each material. For example, four grades of ibuprofen from different suppliers are included (IBU1–IBU4), and three grades of microcrystalline cellulose (MCC1–MCC3) are used to demonstrate excipient influence on tablet formation. Similarly, benzoic acid (BZ) was prepared in-house as primary crystals to study compression behavior in its controlled morphology. The table therefore serves as a reference map for interpreting all subsequent experimental results. Supplementary Table 2 summarizes the key physicochemical and mechanical properties of all materials used in the tablet formulations. These properties are critical for understanding and interpreting the materials' behavior during powder handling, dosing, and compaction within the tablet manufacturing workflow.

Supplementary Table 1: Materials used in this study, including the specific grade and supplier. The Material ID is used across the manuscript to refer to the specific grade of material.

Material ID	Material	Grade	Supplier
**SP**	Paracetamol	Standard 6375	Mallinckrodt
**AS**	Aspirin	Powder	Molekula
**DEX**	Dexamethasone	Powder	Molekula
**GR**	Griseofulvin	Powder	Molekula
**IM**	Indomethacin	Powder	Molekula
**MH**	Metformin Hydrochloride	Powder	Molekula
**BZ**	Benzoic Acid	Primary crystal	Prepared in-house
**LOV**	Lovastatin	Powder	Molekula
**IBU1**	Ibuprofen	CAS 15687-27-1	BLD PharmaTech
**IBU2**	Ibuprofen	Ibuprofen 25	BASF
**IBU3**	Ibuprofen	Ibuprofen 38	BASF
**IBU4**	Ibuprofen	Ibuprofen 50	BASF
**CIPH**	Ciprofloaxcin Hydrochloride	Powder	Molekula
**DOX**	Doxycycline Hyclate	Powder	Molekula
**LVCT**	Levetiracetem	Powder	Molekula
**GF**	Guaifenesin	Powder	Molekula
**ASC**	L-Ascorbic Acid	Powder	Molekula
**MF**	Mefenamic Acid	Powder	Sigma
**MCC1**	Microcrystalline Cellulose	Avicel®PH-101	DuPont Nutrition
**MCC2**	Microcrystalline Cellulose	Avicel®PH-102	DuPont Nutrition
**MCC3**	Microcrystalline Cellulose	Microcel 302	Roquette
**LAC1**	Lactose	Fastflo®316	Foremost Farms USA
**LAC2**	Lactose	Granulac 200M	Meggle pharm
**MAN**	Mannitol	Pearlitol 200 SD	Roquette
**MgSt**	Magnesium Stearate	Hyqual 5712	Mallinckrodt
**CCS**	Croscarmellose Sodium	AcDiSol	FMC International

Supplementary Table 2: Characteristics of materials used in the tablet formulations. Information includes bulk, tapped, and true densities, the flow function coefficient (FFC), and volume-based particle size percentiles (d10, d50, d90), and aspect ratio (s10, s50, s90). FFC is given at a specific consolidation pressure.

**Material ID**	**Density**			**Flowability**	**Volume based distribution**			**Aspect Ratio**		
	Bulk Density (g cm^{-3})	Tapped Density (g cm^{-3})	True Density (g cm^{-3})	FFC	d10 (µm)	d50 (µm)	d90 (µm)	s10 (µm)	s50 (µm)	s90 (µm)
**SP**	0.34	0.55	1.18	1.04 @ 0.30 kPa	24.5	88.2	205.0	0.289	0.561	0.826
**AS**	0.77	0.86	1.39	9.29 @ 0.81 kPa	68.8	148.2	322.7	0.471	0.685	0.873
**DM**	0.32	0.41	1.39	1.34 @ 0.80 kPa	9.7	25.2	176.6	0.484	0.698	0.880
**GR**	0.36	0.52	1.58	2.00 @ 0.29 kPa	14.5	29.7	99.0	0.474	0.700	0.886
**IM**	0.36	0.52	1.58	2.98 @ 0.29 kPa	45.0	93.0	165.2	0.477	0.705	0.885
**MH**	0.67	0.76	1.35	3.91 @ 0.80 kPa	22.2	192.0	352.5	0.410	0.655	0.877
**BZ**	0.28	-	1.30	-	23.2	41.6	58.8	0.465	0.674	0.861
**MF**	0.62	0.45	1.26	-	64.7	153.0	268.1	0.388	0.657	0.859
**LOV**	0.59	0.43	1.11	-	28.5	62.2	108.4	0.351	0.575	0.815
**IBU1**	0.44	0.52	1.12	5.95 @ 0.80 kPa	51.46	94.44	125	0.576	0.732	0.891
**IBU2**	0.46	0.37	1.16	2.70 @ 0.80 kPa	16.44	30.33	50.83	0.722	0.831	0.93
**IBU3**	0.48	0.40	1.12	1.94 @ 0.80 kPa	17.32	39.06	70.79	0.628	0.754	0.894
**IBU4**	0.53	0.46	1.11	2.47 @ 0.80 kPa	17.9	48.96	77.05	0.642	0.768	0.904
**CIPH**	0.68	0.53	1.32	2.31 @ 0.80 kPa	28.7	67.6	124.2	0.374	0.637	0.866
**DOX**	0.70	0.52	1.47	1.67 @ 0.80 kPa	30.6	75.1	189.7	0.354	0.598	0.848
**LVCT**	0.69	0.58	1.29	2.12 @ 0.80 kPa	55.7	117.4	209.0	0.368	0.608	0.847
**GF**	0.61	0.45	1.34	1.71 @ 0.80 kPa	47.9	102.9	182.8	0.420	0.706	0.901
**ASC**	1.14	0.98	1.70	4.33 @ 0.80 kPa	43.4	120.7	261.6	0.455	0.700	0.890
**MCC1**	0.33	0.45	1.56	5.85 @ 0.80 kPa	27.5	53.5	104.0	0.333	0.577	0.833
**MCC2**	0.34	0.44	1.56	3.91 @ 0.79 kPa	44.5	86.3	153.2	0.347	0.597	0.833
**MCC3**	0.45	0.51	1.53	4.44 @ 0.80 kPa	25.5	62.4	152.0	0.407	0.653	0.860
**LAC1**	0.63	0.74	1.54	5.55 @ 1.06 kPa	55.1	104.7	188.4	0.494	0.715	0.911
**LAC2**	0.52	0.76	1.55	1.56 @ 0.80 kPa	22.4	59.7	107.3	0.499	0.706	0.881
**MAN**	0.54	0.59	1.48	12.07 @ 1.06 kPa	68.8	115.1	191.1	0.047	0.679	0.869
**MgSt**	0.28	0.35	1.30	1.44 @ 0.30 kPa	15.7	40.2	76.6	0.422	0.644	0.857
**CCS**	0.54	0.74	1.60	3.60 @ 0.80 kPa	30.4	51.6	82.1	0.372	0.613	0.832

2 Hybrid System of Models

2.1 Data Summary

Supplementary Table 3: Summary of tablet data reported per API and placebo data set. “T” and “V” indicates the use of the data for the “Training” or “Validation” of the process models, respectively. The “Min” and “Max” indicates the minimum and maximum values in the respective data set.

Material ID	Train./ Val.	Drug loading		Porosity		Tensile Strength (MPa)		Compression Pressure (MPa)	
		**Min**	**Max**	**Min**	**Max**	**Min**	**Max**	**Min**	**Max**
**Placebo**	T	0.00	0.00	0.03	0.39	0.03	13.92	6	565
**BZ**	T	0.35	0.35	0.06	0.81	0.87	4.52	55	398
**LOV**	T	1.00	1.00	0.06	0.43	0.07	2.29	12	326
**IBU1**	T	0.01	0.53	0.03	0.36	0.04	3.09	12	369
**MF**	T	0.05	0.46	0.06	0.38	0.03	4.48	14	368
**SP**	V	0.01	0.32	0.04	0.22	0.04	8.18	12	489
**GR**	V	0.20	0.20	0.11	0.15	2.55	4.26	144	258
**AS**	V	0.20	0.20	0.08	0.14	1.01	4.11	71	450
**ASC**	V	0.20	0.35	0.07	0.22	0.63	3.36	64	472
**CIPH**	V	0.20	0.35	0.07	0.20	1.11	5.13	64	394
**DOX**	V	0.20	0.35	0.07	0.21	1.35	5.14	64	318
**GF**	V	0.20	0.20	0.09	0.17	1.18	2.84	131	450
**IM**	V	0.20	0.20	0.13	0.21	2.06	4.13	91	206
**LVCT**	V	0.20	0.35	0.07	0.24	0.40	3.22	64	319
**MH**	V	0.20	0.20	0.10	0.26	0.69	3.45	70	450
**DEX**	V	0.20	0.20	0.09	0.23	2.02	6.63	70	434
**IBU2**	V	0.06	0.32	0.23	0.19	0.80	5.28	74	485
**IBU3**	V	0.06	0.32	0.04	0.20	0.79	5.12	74	483
**IBU4**	V	0.06	0.32	0.03	0.20	0.65	5.12	75	491

Supplementary Table 4: List of crystal structure and Particle Informatics descriptors used for data-driven prediction of porosity and tensile strength.

Material ID	Packing Coefficient	Average Hydrogen Bond Donor Density	Average Hydrogen Bond Acceptor Density	Average Rugosity	Average Surface Charge	Short-to-Medium Axis Length Ratio	Medium-to-Long Axis Length Ratio	Hydrogen Bond Dimensionality
**SP**	0.727	0.046	0.043	1.826	-0.196	0.868	0.835	2
**GR**	0.703	0	0.069	1.659	-0.356	1	0.554	-1
**IBU**	0.676	0.009	0.018	1.768	-0.212	0.424	0.864	0
**IM**	0.703	0.012	0.055	1.643	-0.479	0.697	0.425	0
**BZ**	0.713	0.026	0.053	1.265	-0.302	0.478	0.927	0
**LOV**	0.702	0.005	0.033	1.547	-0.058	0.864	0.467	1
**MF**	0.691	0.040	0.061	1.729	-0.372	0.454	0.838	0
**DEX**	0.694	0.026	0.048	1.934	-1.080	0.847	0.768	3
**MH**	0.713	0.026	0.053	1.265	-0.302	0.478	0.927	0
**LVCT**	0.668	0.129	0.137	1.768	-0.093	0.697	0.756	0
**GF**	0.671	0.136	0.149	1.659	-0.105	0.826	0.729	0
**DOX**	0.661	0.127	0.069	0.142	-0.389	0.837	0.721	0
**AS**	0.715	0.083	0.086	1.365	-0.295	0.692	0.584	0
**ASC**	0.673	0.143	0.158	1.643	-0.212	0.812	0.734	0
**CIPH**	0.662	0.132	0.147	1.128	-0.097	0.828	0.742	0

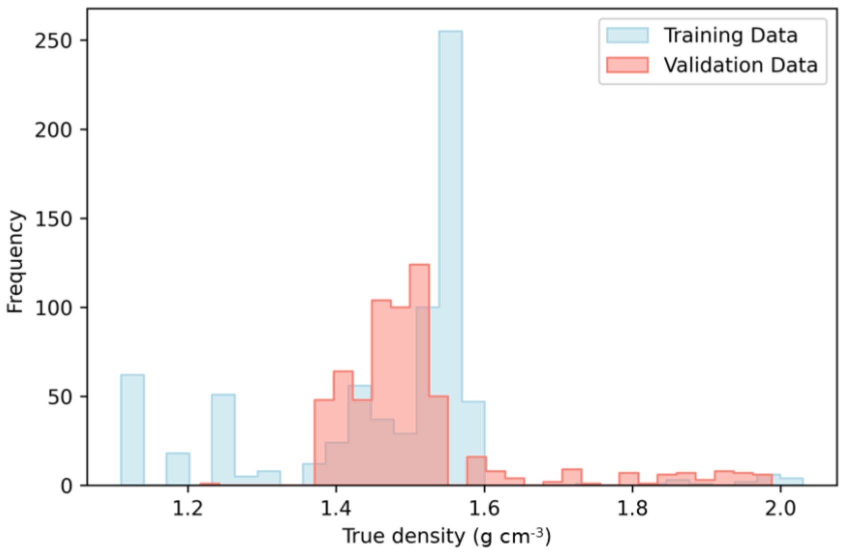

Supplementary Figure 1: Frequency histogram of true density values of training and validation data. Source data are provided as a Source Data file.

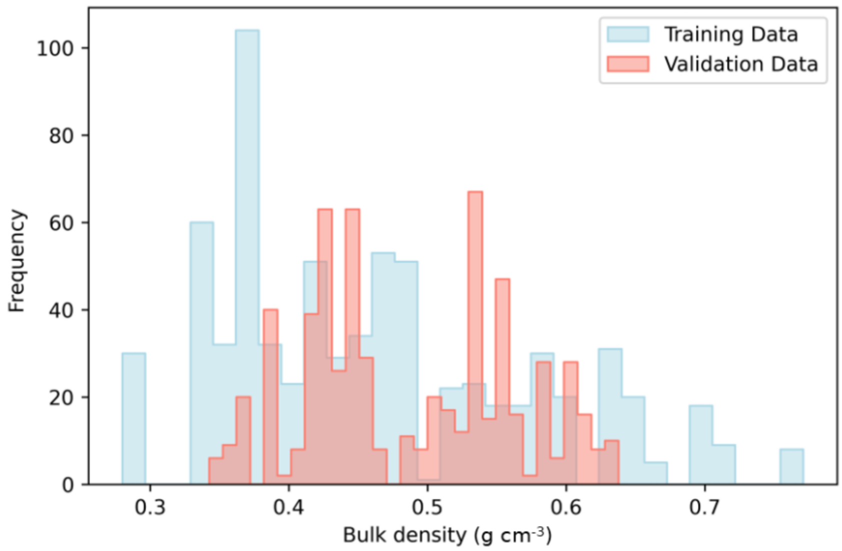

Supplementary Figure 2: Frequency histogram of bulk density values of training and validation data. Source data are provided as a Source Data file.

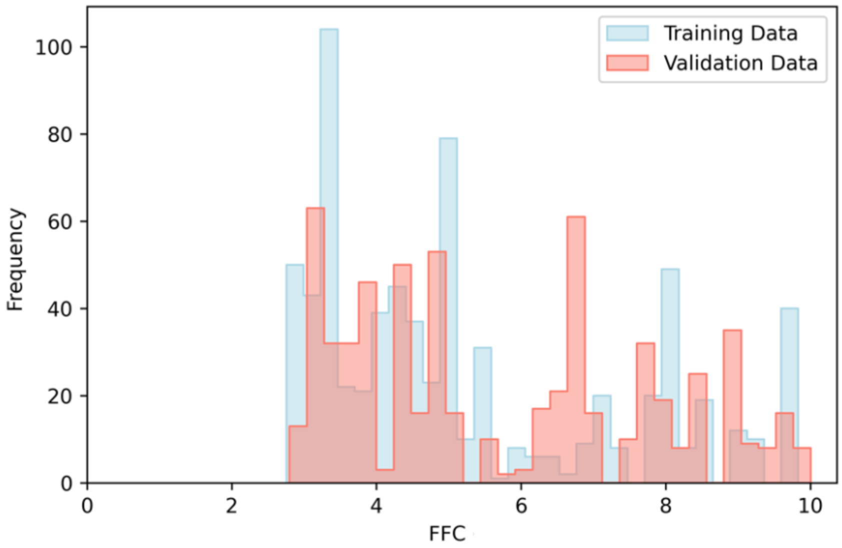

Supplementary Figure 3: Frequency histogram of FFC values (at 1.6 KPa) of training and validation data. Source data are provided as a Source Data file.

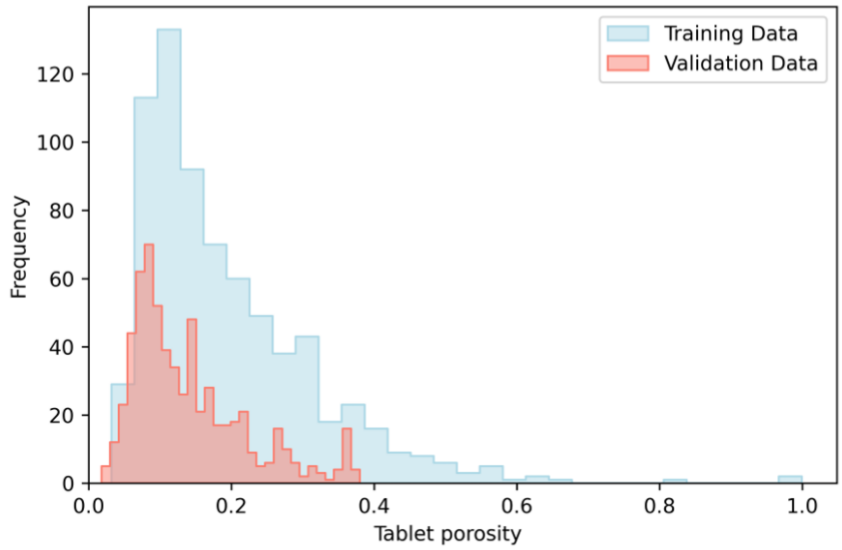

Supplementary Figure 4: Frequency histogram of tablet porosity values of training and validation data. Source data are provided as a Source Data file.

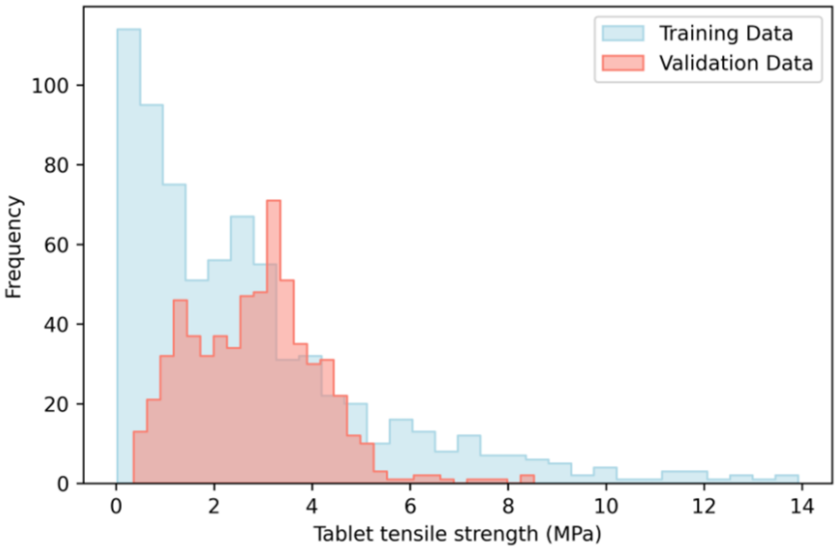

Supplementary Figure 5: Frequency histogram of tablet tensile strength values of training and validation data. Source data are provided as a Source Data file.

2.2 Validation of FFC mixture model

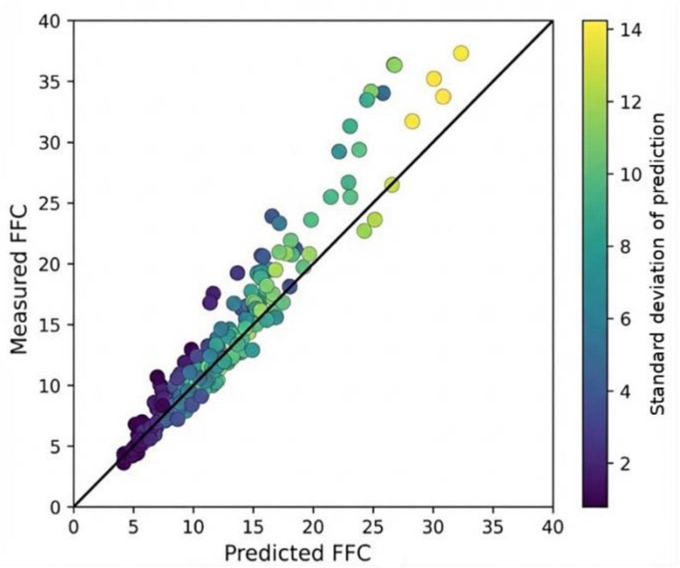

Supplementary Figure 6: Prediction performance of FFC mixture model developed by Salehian, Moores [1]. The model was trained with 61 mixture data (i.e., 305 data points) while 44 mixtures (i.e., 220 data points as shown in the figure) were kept as the test dataset to validate the prediction accuracy. The R^2 and $RMSE$ are 0.93 and 0.013, respectively. The colour bar shows the standard deviation of the predicted data points. Source data are provided as a Source Data file.

2.3 Development and validation of process models

To ensure the reliability and robustness of the process models, a systematic data quality control pipeline was implemented after ingesting the original dataset to ensure the physical validity of the training and validation data (Supplementary Figure 7). The pipeline enforces data integrity criteria through multiple automated stages:

- Data cleaning: The dataset is screened for logging errors, removing entries with missing values, or non-physical values (negative, zero, or NaN) for compression pressure, porosity, and tensile strength.

- Empirical quality check: To filter out the outlier data resulting from measurements of tablets with defects or data collection errors such as over-compression[2], capping[3], lamination[4], or data collection issues, the compressibility and compactability profiles of each formulation are fitted to the Kawakita[5] and Ryshkewitch-Duckworth[6] models, respectively. A user-defined goodness-of-fit threshold ($R^2 < 0.7$ in this study) is applied. Formulations failing to meet this conservative threshold are flagged as low-fit outliers and excluded from the original dataset to ensure the models are developed and validated using only physically valid data.

At the initial stage of model training and validation (Version 1), all input parameters except for informatics descriptors (i.e.,, parameters in Supplementary Table 4) were utilised to train data-driven models using three approaches: Deep neural network (DNN), Random forest (RF), and Support vector regression (SVR). These modelling approaches were selected to encompass a diverse range of ML-based modelling techniques, where DNN represent deep learning-based approaches capable of capturing complex nonlinear relationships[7], RF is an ensemble-based method known for its robustness and interpretability[8], and SVR is a kernel-based approach well-suited for capturing intricate patterns in smaller datasets[9]. This allowed for a comparative analysis of their predictive performance.

The predictive performance of three modelling approaches was compared, showing the superior performance of the Deep neural networks (DNNs) in predicting the porosity and tensile strength of tablets containing previously unseen APIs. This comparison was initially performed without using the particle informatic descriptors (Supplementary Figure 8, Supplementary Figure 9, and Supplementary Figure 10) followed by using all parameters in Table 1 as input parameters for process models.

Given the DNN's superior performance over Random forest (RF) and Support vector regression (SVR), the DNNs were retrained incorporating all parameters listed in Table 1, including the crystallographic and particle informatics descriptors [10, 11], to examine the impact of considering API crystal structure on prediction accuracy (Version 2). The comparative analysis of prediction performance between the initial version (Version 1 in Supplementary Figure 8) and expanded input features (Version 2 in Figure 3) demonstrates a marginal improvement in the accuracy of the DNNs following the inclusion of CSD particle data in the input parameters (Supplementary Table 5). The marginal contribution analysis indicates that the proposed modelling framework is predominantly influenced by particle-level attributes rather than molecular-level crystal structure, prioritising the optimisation of particle properties and processing conditions to enhance tablet manufacturability. This enhanced version of the models including the CSD particle data is utilised in the formulation optimisation framework.

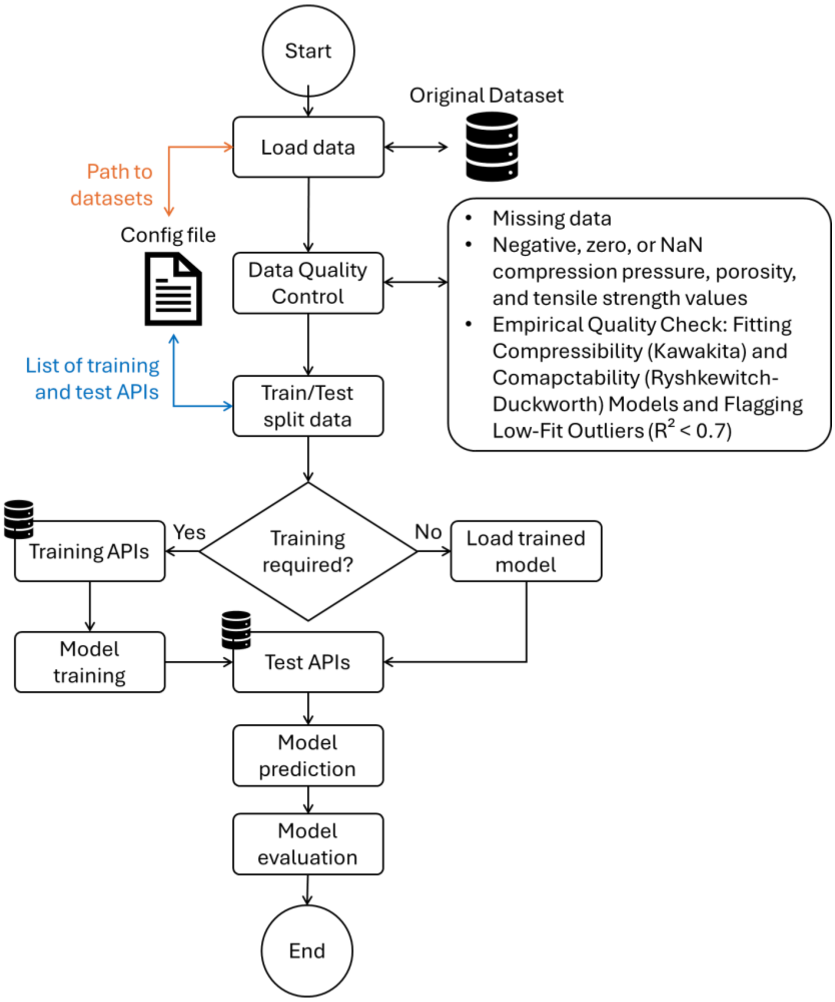

Supplementary Figure 7: Schematic representation of the automated data ingestion, quality control, and training/validation framework for process models. Source data are provided as a Source Data file.

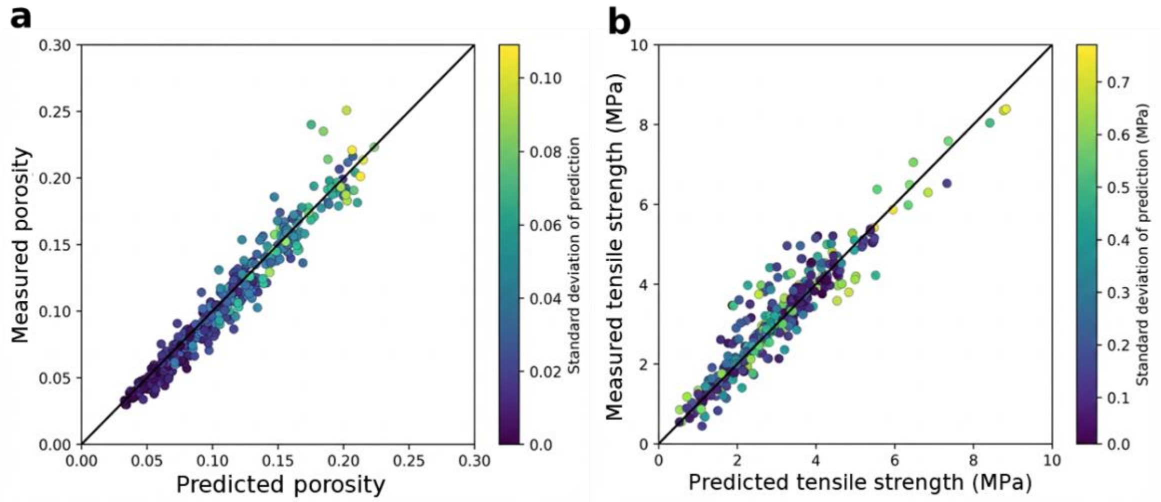

Supplementary Figure 8: Prediction performance of **a** porosity and **b** tensile strength using ensemble of DNNs based on the validation data. Particle informatics descriptors are excluded from input parameters. The colour bars show the estimated standard deviation of predicted test data. Source data are provided as a Source Data file.

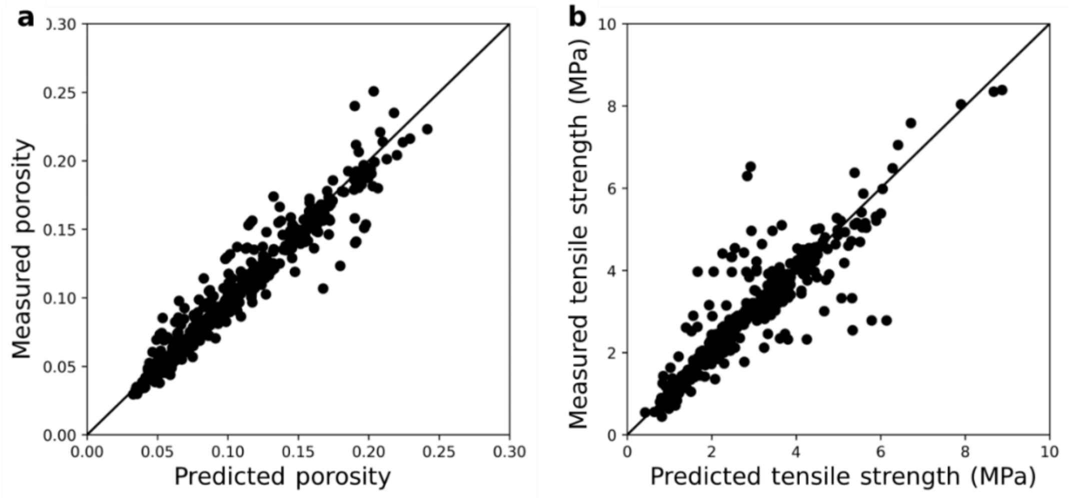

Supplementary Figure 9: Prediction performance of **a** porosity and **b** tensile strength using RF based on the validation data. Particle informatics descriptors are excluded from input parameters. Source data are provided as a Source Data file.

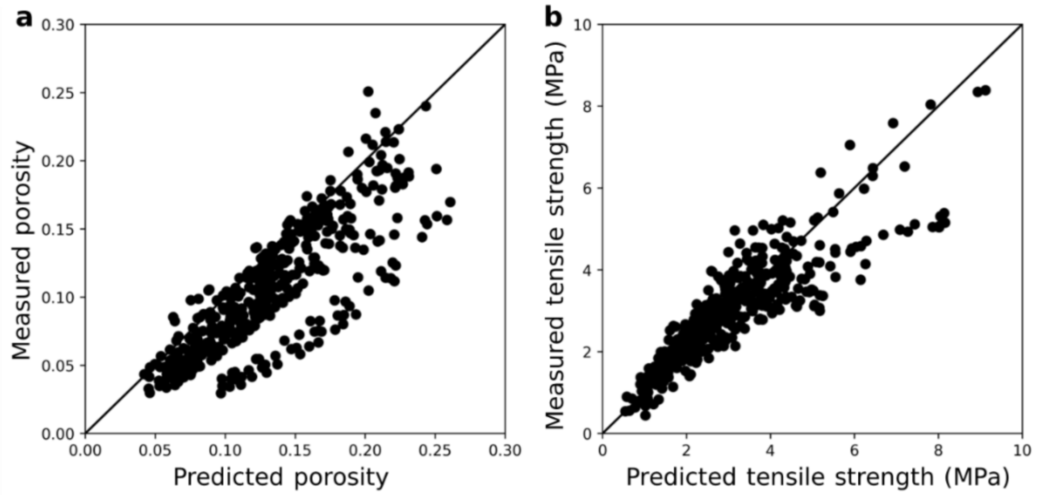

Supplementary Figure 10: Prediction performance of **a** porosity and **b** tensile strength using SVM based on the validation data. Particle informatics descriptors are excluded from input parameters. Source data are provided as a Source Data file.

2.4 Model performance summary

Supplementary Table 5: Validation accuracy (R^2 and RMSE) of process models with and without including CSD parameters.

	**Without CSD parameters (Version 1)**				**With CSD parameters (Version 2)**			
	**Porosity**		**Tensile Strength**		**Porosity**		**Tensile Strength**	
	R^2	RMSE	R^2	RMSE (MPa)	R^2	RMSE	R^2	RMSE (MPa)
**DNN**	0.90	0.02	0.86	0.61	0.90	0.01	0.89	0.40
**RF**	0.89	0.03	0.74	0.80	-	-	-	-
**SVM**	0.64	0.07	0.67	0.88	-	-	-	-

Supplementary Table 6: Validation accuracy of DNN model with CSD parameters per validation API.

API	Porosity		Tensile Strength	
	R^2	RMSE (MPa)	R^2	RMSE (MPa)
**SP**	0.89	0.01	0.86	0.30
**GR**	0.91	0.00	0.98	0.08
**AS**	0.85	0.02	0.80	0.49
**ASC**	0.98	0.00	0.93	0.17
**CIPH**	0.99	0.00	0.90	0.93
**DOX**	0.90	0.01	0.88	0.86
**GF**	0.93	0.01	0.93	0.19
**IM**	0.85	0.01	0.98	0.12
**LVCT**	0.84	0.02	0.85	0.51
**MH**	0.79	0.02	0.87	0.29
**DEX**	1.00	0.00	0.86	0.56
**Average**	**0.90**	**0.01**	**0.89**	**0.40**

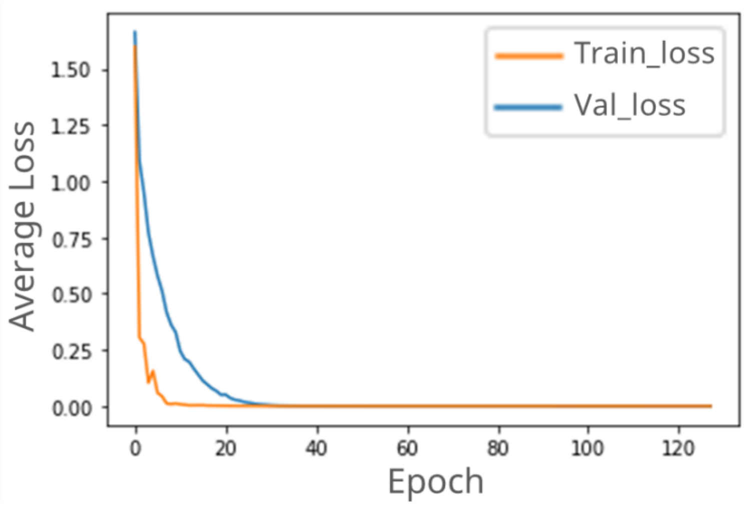

Supplementary Figure 11: Average training and validation loss of ensemble of porosity models (Version 2 with CSD particle informatics). Early stopping callback is used to prevent overfitting and optimise training time. Source data are provided as a Source Data file.

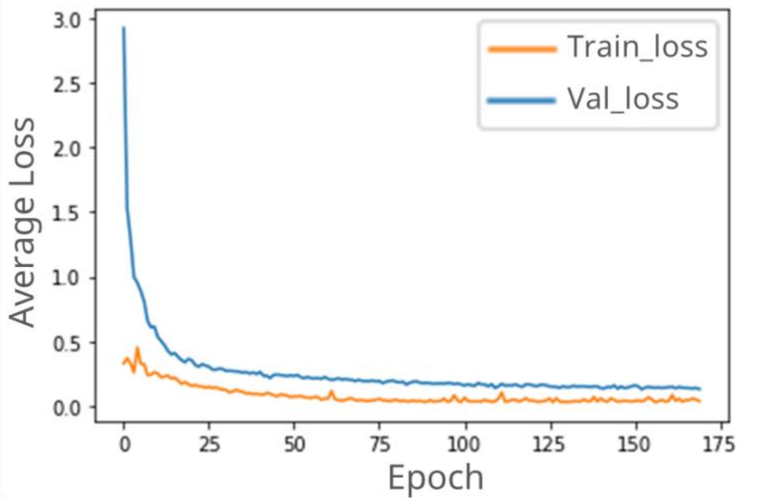

Supplementary Figure 12: Average training and validation loss of ensemble of tensile strength models (Version 2 with CSD particle informatics). Early stopping callback is used to prevent overfitting and optimise training time. Source data are provided as a Source Data file.

2.5 Relative feature importance analysis

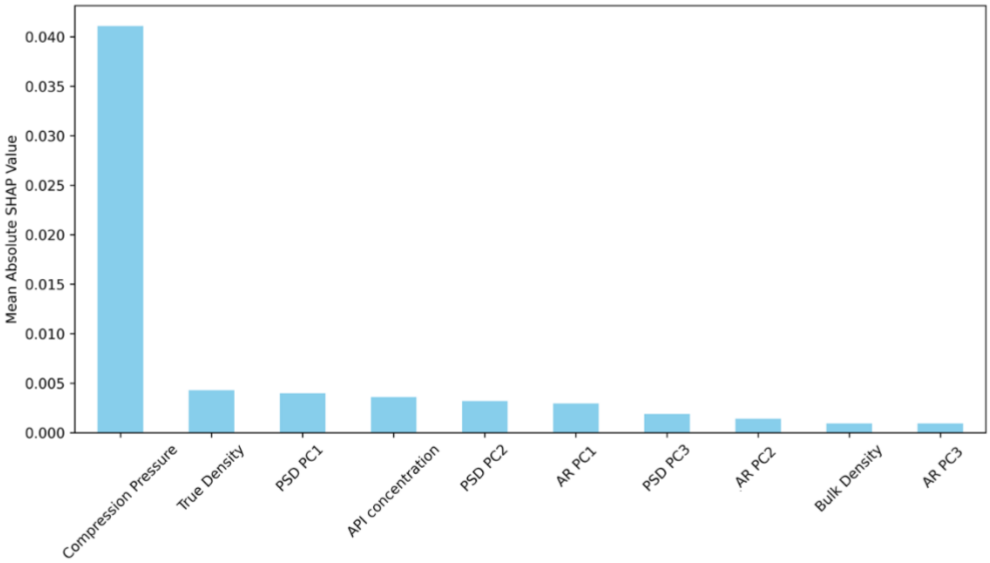

Supplementary Figure 13: Feature importance analysis for the porosity model (Version 2 with CSD particle informatics). The ten features with highest impact based on their mean absolute SHAP values are ranked visualised. Source data are provided as a Source Data file.

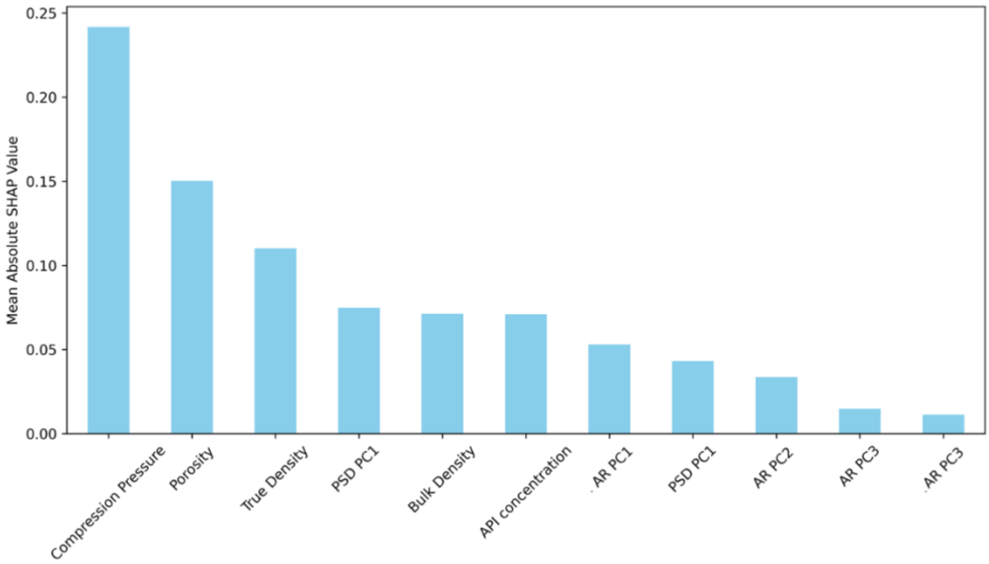

Supplementary Figure 14: Feature importance analysis for the tensile strength model (Version 2 with CSD particle informatics). The ten features with highest impact based on their mean absolute SHAP values are ranked visualised.

3 Tableting data factory

3.1 Make & test

3.1.1 List of instruments

Supplementary Table 7: List of the instruments used in the tableting data factory and their digital interfaces.

Process	Instrument	Digital interface
Powder dosing	DEC Flex PTS	Profinet
NIR spectroscopy	VIAVI Micro NIR	OPC UA
Powder compaction	MEDELPHARM STYL'One Nano	Websocket
Tablet tester	Sotax AT50	OPC DA
Robot 1	Universal UR5e	TCP/IP
Robot 2	Kuka LBR iiwa 14	UDP
Weighing balance	Cole-Parmer	Serial Communication

3.1.2 Powder transportation unit (TU)

The custom-built transportation unit consists of a tube that contains powder for transport and analysis as shown in Supplementary Figure 15. The tube's inlet has a width of 20 mm, allowing sufficient space for powder dosing. The outlet, however, is 8 mm wide, which is just below the 9 mm diameter of the tablet press die where the powder will be discharged. Powder flow is regulated by a sliding gate driven by an electronic solenoid, while an electric vibrator ensures smooth powder flow through the tube. A clear sapphire disk, 10 mm in diameter, is placed above the sliding gate to allow for NIR scanning. A robotic arm (R1) performs pick-and-place tasks by holding the transportation unit from the back and supplying power to the vibrator and solenoid.

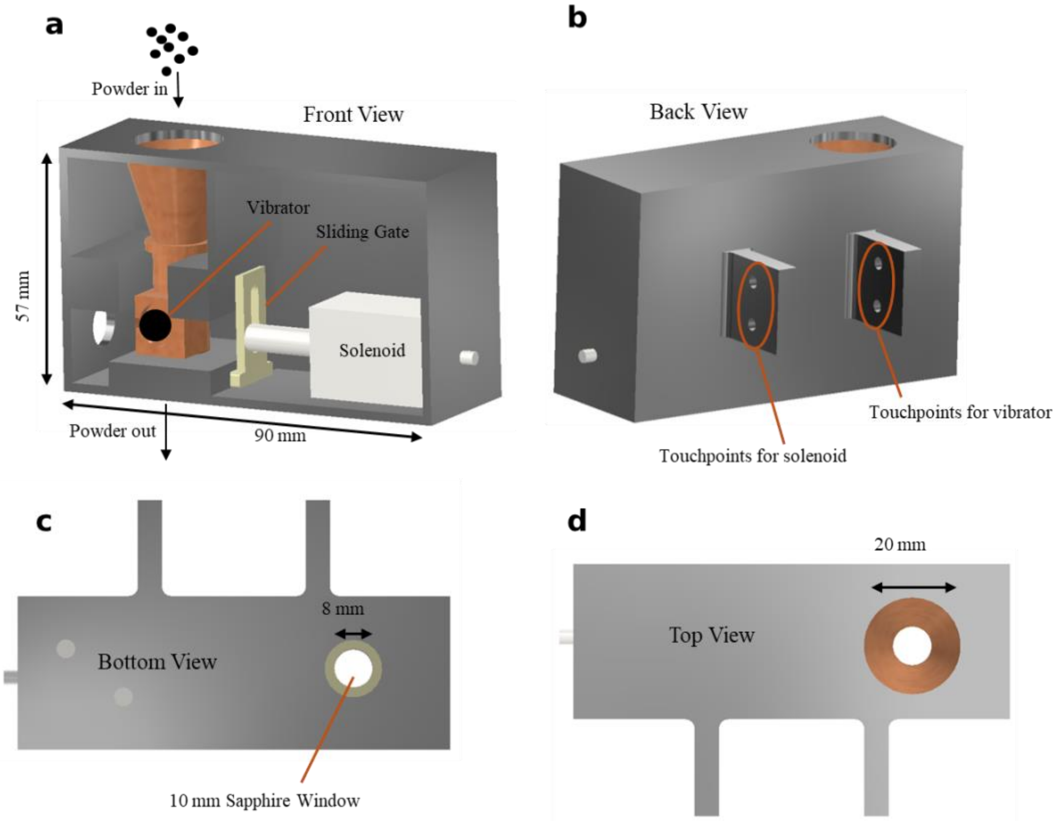

Supplementary Figure 15: Design of TU. **a** Front side of the TU; **b** Back side of the TU; **c** Bottom side of the TU; **d** Top side of the TU.

3.1.3 Robotic fingers

Supplementary Figure 16 shows the design of customised 3D-printed fingers mounted on R1, which enable both the picking and placing of the TU as well as the provision of electrical energy through touchpoints. Supplementary Figure 17 illustrates the design of customized fingers for R2, which are specifically made for tablet transportation. The fingers are engineered to securely pick up tablets from both horizontal and vertical orientations, ensuring that the tablets remain securely held without slipping.

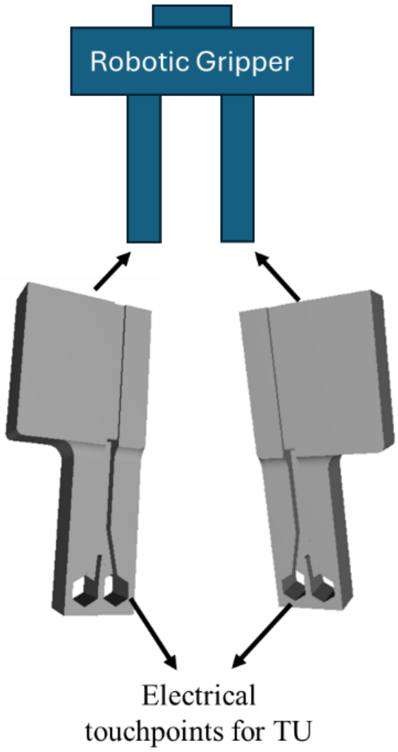

Supplementary Figure 16: Fingers for the gripper of R1 to hold TU and to provide electrical power.

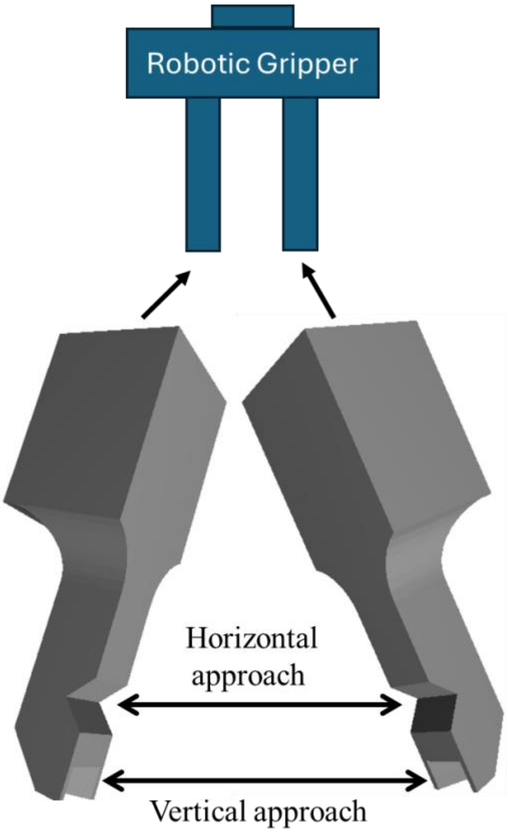

Supplementary Figure 17: Fingers for the gripper of R2 to pick and place tablets.

3.1.4 Automated tablet separator (ATS)

The automated tablet separator (ATS) (Supplementary Figure 18) is designed to reliably distinguish and route damaged tablets from intact ones as part of the self-driving manufacturing workflow. This separation step is essential to prevent fractured or compromised tablets, typically resulting from destructive testing methods, from entering downstream stages such as storage of samples. The ATS consists of two outlet channels and a central, servo-actuated barrier that controls the routing of each tablet. As tablets exit the testing station, the system identifies whether the preceding test was destructive or non-destructive. Based on this information, the microcontroller sends a command to the servo motor to position the barrier either left or right, thereby directing the tablet into the appropriate channel. This logic enables the separator to function without requiring additional sensing hardware, minimizing system complexity while maintaining high reliability. For intact tablets, the assigned outlet channel incorporates a linear solenoid actuator that gently advances the tablet forward once it is detected in position. This motion ensures that the tablet is presented at a consistent orientation and distance, facilitating rapid and accurate grasping by the robotic gripper. Damaged tablets, in contrast, are diverted into a separate collection channel for disposal or off-line inspection. By isolating tablets based on the type of test applied (destructive vs. non-destructive), the ATS prevents cross-contamination of data, maintains the integrity of the process flow, and enables fully automated progression from compaction to characterization. This unit therefore plays a critical role in preserving traceability and ensuring that only valid, structurally sound tablets are used for subsequent analyses within the self-driving experimental framework.

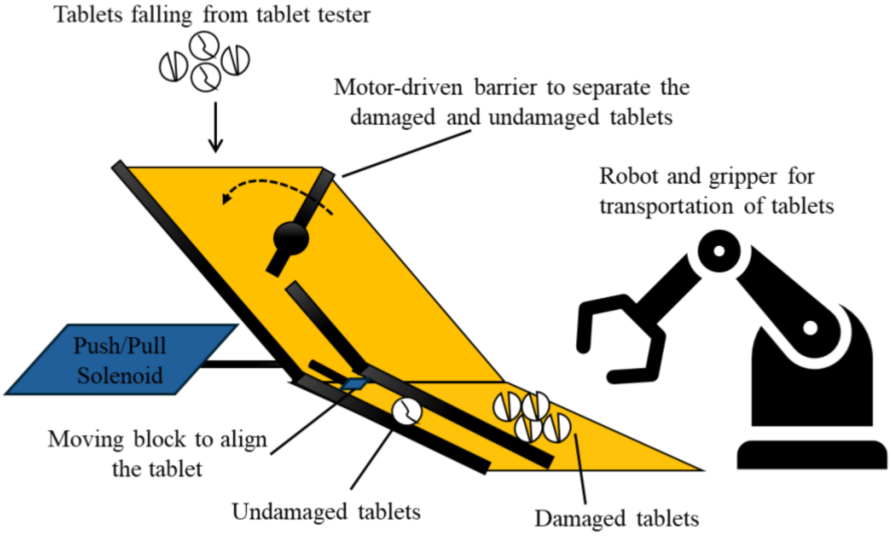

Supplementary Figure 18: Tablet separator for damaged and undamaged tablets.

3.1.5 Customised cleaning unit (CU) for TU

The cleaning unit (CU) is designed to remove powder residue from the TU by sweeping the front, top, and bottom surfaces after each compaction cycle. Effective cleaning is essential in automated tablet manufacturing, as residual powder can interfere with die filling, affect tablet weight consistency, and compromise the performance of downstream modules such as the testing unit or robotic handling system. The CU is integrated with a high-powered vacuum system, which provides the suction force necessary to extract fine particles from the TU surfaces. The vacuum is driven by an external control unit that interfaces directly with the supervisory control framework. As illustrated in Supplementary Figure 19, the CU incorporates a custom-designed cleaning head that aligns with the geometry of the TU, ensuring that all critical surfaces, including corners and recesses, are properly cleared of powder buildup. All

operations of the CU, including activation timing, duration of cleaning, and coordination with the TU and upstream compaction module, are fully automated through the LabVIEW-based supervisory control system. This real-time control allows the CU to be triggered immediately after tablet ejection or following specific events where powder generation is expected, thereby preventing accumulation and reducing manual intervention. By maintaining the cleanliness of the TU consistently and reproducibly, the CU enhances system reliability, reduces cross-contamination risk between experiments, and supports long-duration autonomous operation of the self-driving platform.

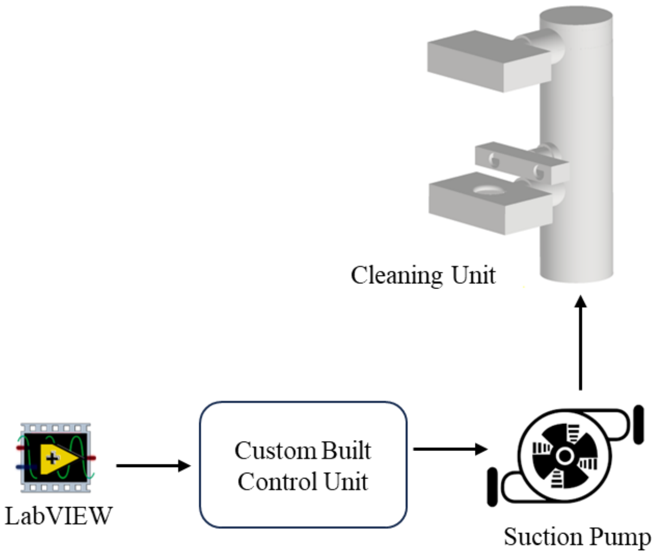

Supplementary Figure 19: Design of customised CU for the cleaning of TU.

3.1.6 Process analysis with near-infrared (NIR) spectroscopy

NIR spectroscopy is a rapid, non-destructive analytical technique and widely used for process monitoring and control in pharmaceutical applications. NIR spectra capture key chemical and physical properties of samples, providing valuable information about many critical parameters, such as blend homogeneity.

3.1.7 Orchestration system

Supplementary Figure 20 shows how all the devices and instruments are digitally integrated from end to end with supervisory control unit. The dosing unit has its own dedicated programmable logic controller (PLC) and does not require additional software for control. It communicates with the orchestration system via the Profinet protocol, with each PLC tag (parameters/variables) predefined in the orchestration system for control purposes. The weighing balance is integrated with the orchestration system through serial communication. The orchestration system sends a read command to the balance, which then returns the current weight of the dose. The micro NIR instrument is controlled via proprietary software (VIAVI micro NIR), which offers remote access through the OPC interface. All configurations and method-related information are set up in this software. Once the method is created, the dark, reference, and sample scans can be managed through the orchestration system, with spectra acquired via OPC communication protocol. The tablet press is operated using proprietary Alix software, which communicates with a remote computer through a WebSocket interface. The tablet press's communication interface allows for greater flexibility in adjusting parameters remotely without relying on its proprietary software. Similarly, the tablet tester is managed through Q-doc, a proprietary software installed on a device-specific laptop. All necessary

parameters for creating destructive and non-destructive profiles are predefined in Q-doc, which can send and receive data remotely through an OPC interface. The R1 and R2 robots are integrated with the orchestration system via TCP and UDP socket interfaces, respectively. R1's control system uses block-based programming, where each block represents a specific function, while R2 is programmed using Java-based scripting. Both robots move between instruments based on pre-defined waypoints, with their movements triggered by instructions from the orchestration system. Finally, all the bespoke units, including TU, CU and TS are integrated with orchestration system through embedded controller and serial communication. The embedded controller receives the specific instruction from orchestration system and then performs the actions on bespoke units.

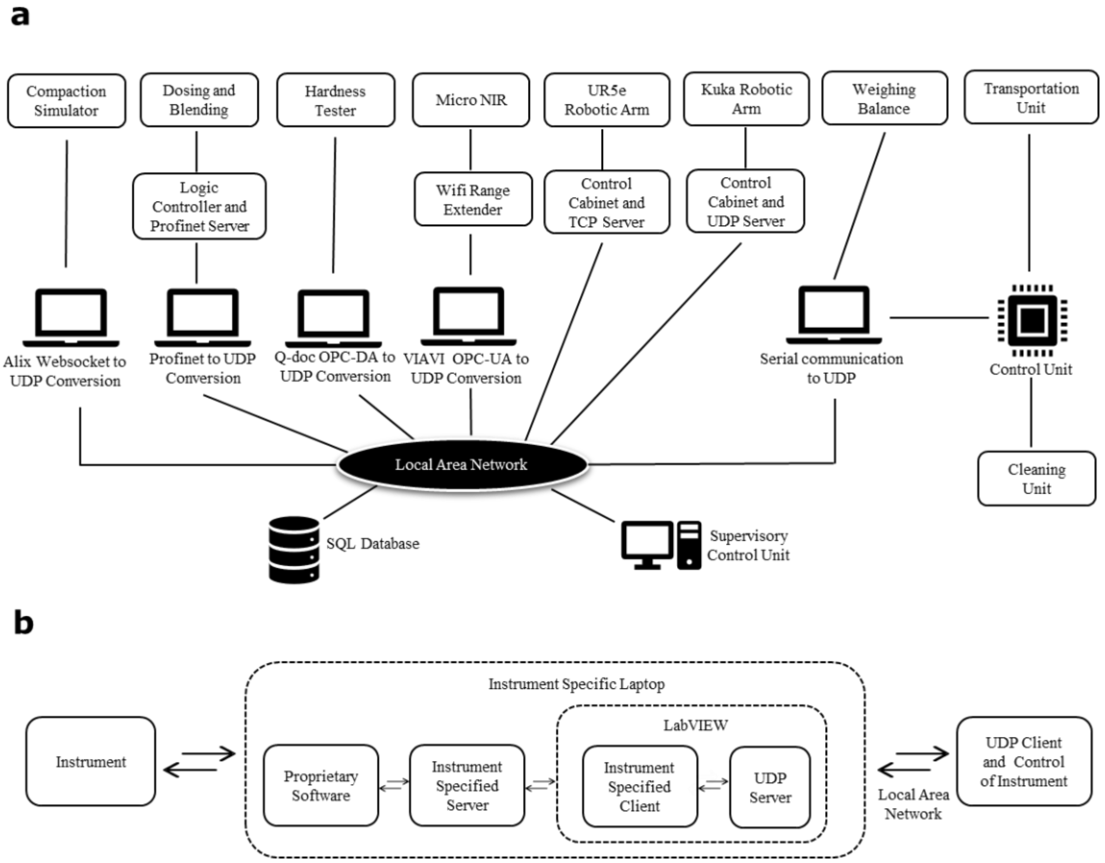

Supplementary Figure 20: Digital integration of instruments with orchestration system. **a** Explains the communication protocols used for all the instruments and devices with orchestration system through local area network. **b** Explains how a layer of standardized UDP interface is added around each of instrument.

3.1.8 Validation of tableting data factory

Five distinct ternary blends, exhibiting varying concentrations of paracetamol at 1%, 5%, 10%, 15%, and 20 wt.%, respectively, were prepared as listed in Supplementary Table 8. The paracetamol was mixed with lactose using a laboratory powder blender (PharmaTech Multiblend MB015). The blending process was performed at a blender speed of 20 rpm and an agitator speed of 200 rpm over a duration of 20 min to ensure homogeneity. In addition to the formulation process, all blends were lubricated with 1 wt.% of magnesium stearate, a procedure implemented to improve the flow properties of the blends. This lubrication step was executed by allowing the blend to mix for an additional 5 min, thus ensuring a uniform distribution of the lubricant without overlubricating the mixture. In our study, we also prepared nine quaternary placebo blends, each planned to include a fixed concentration of the disintegrating agent, croscarmellose sodium (5 wt.%). The 1% blends used a staged blending procedure where

the API was initially blended with a smaller volume of filler before being introduced to the main bulk to reduce the presence of agglomerated API. These blends were designed to have varied of filler ratios to broaden the knowledge space. The filler combinations utilised included microcrystalline cellulose and dibasic calcium phosphate, mannitol, and lactose, as well as mannitol and dibasic calcium phosphate. Consistency was maintained in the formulation process across the blends, employing the same laboratory powder blender (PharmaTech Multiblend MB015) with fixed settings of a 20-rpm blender speed and a 200-rpm agitator speed, operating over a duration of 20 min. As with the paracetamol blends, each placebo blend was lubricated with 1 wt.% of magnesium stearate to promote the blend characteristics. A detailed overview of the precise formulations and the corresponding concentrations (wt.%) of each filler in each blend prepared are illustrated in Supplementary Table 8.

Supplementary Table 8: Composition of the tablet blends used for validation of tableting data factory. The data in the table represents the percentage weight by weight (wt.%) of each ingredient in the formulation. Each entry in the table specifies the unique identifier of API and excipients followed by the concentration.

Blend ID	API (wt.%)	Disintegrant (wt.%)	Filler/Binder 1 (wt.%)	Filler/Binder 2 (wt.%)	Lubricant (wt.%)
**B1**	SP (1%)	-	LAC1 (98%)	-	MgSt (1%)
**B2**	SP (5%)	-	LAC1 (94%)	-	MgSt (1%)
**B3**	SP (10%)	-	LAC1 (89%)	-	MgSt (1%)
**B4**	SP (15%)	-	LAC1 (84%)	-	MgSt (1%)
**B5**	SP (20%)	-	LAC1 (79%)	-	MgSt (1%)
**B6**	-	CCS (5%)	MAN (30%)	DCPA (64%)	MgSt (1%)
**B7**	-	CCS (5%)	MAN (30%)	LAC1 (64%)	MgSt (1%)
**B8**	-	CCS (5%)	MAN (64%)	LAC1 (30%)	MgSt (1%)
**B9**	-	CCS (5%)	MCC (30%)	DCPA (64%)	MgSt (1%)
**B10**	-	CCS (5%)	MCC (64%)	DCPA (30%)	MgSt (1%)

Supplementary Figure 21 shows data points for multiple tablet formulations labelled B1 through B10 and different dose weights. The powder obtained on x-axis represents the measured weight of powder obtained from dosing unit before compression, and the y-axis represents the resulting tablet weight after compression. The diagonal line represents the ideal condition where tablet weight exactly matches powder weight, indicating zero material loss or compression effect. Data points clustered below the line suggest some weight loss during transportation. The spread of points across different formulations and dose weights highlights variability in tablet production consistency, with some formulations and dose weights achieving weights closer to the powder input, while others deviate more.

Supplementary Figure 22a shows that powder loss during transportation, encompassing powder dosing, adherence to the TU tube, and spillage when opening the TU gate, remains consistently below 22 mg for various formulations. Despite the challenges posed by poor flowability in some Paracetamol formulations, TU maintains a consistent powder loss across various formulations. The formulations B6 and B8 exhibit the least powder loss due to their suboptimal flowability, resulting in minimal spillage during powder dosing and release from TU. Supplementary Figure 22b indicates that there is no significant influence of dose weights on powder loss, with the powder loss consistently staying below 22 mg for all dose weights.

In Supplementary Figure 23a and Supplementary Figure 23b, average and the repeatability of both powder dose and tablet weight is depicted for different formulations. The standard deviation in the powder obtained from the dosing unit directly corresponds to variations in

tablet weight data. As shown in Supplementary Figure 23c and Supplementary Figure 23d, average and the relative standard deviation in the powder data and tablet weight decreases as the dose weight increases, indicating that the dosing system performs more effectively with higher dose weights.

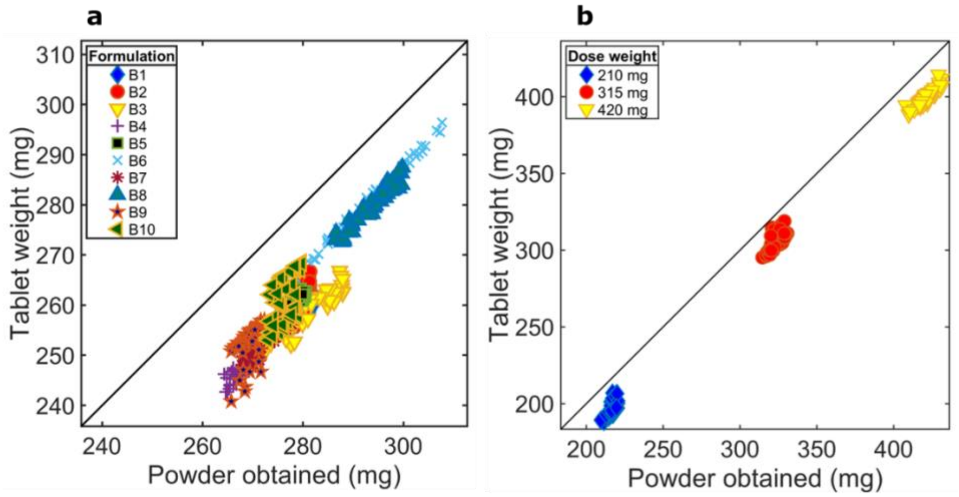

Supplementary Figure 21: Comparison of tablet weight with powder obtained with **a** different formulations and **b** different dose weights. Source data are provided as a Source Data file.

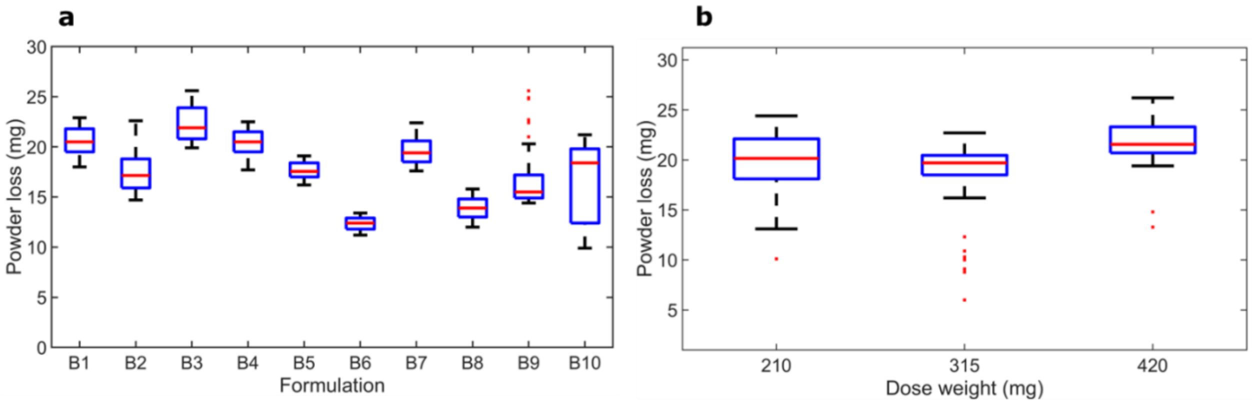

Supplementary Figure 22: Powder loss during dosing, transportation and release across formulations and dose weights. **a** Powder loss for different formulations and, **b** different dose weights. Boxplots are based on 50 independent measurements per condition; the centre line denotes the median, boxes indicate the interquartile range (IQR), whiskers represent the data range excluding outliers, and points denote outliers. Source data are provided in the Source Data file.

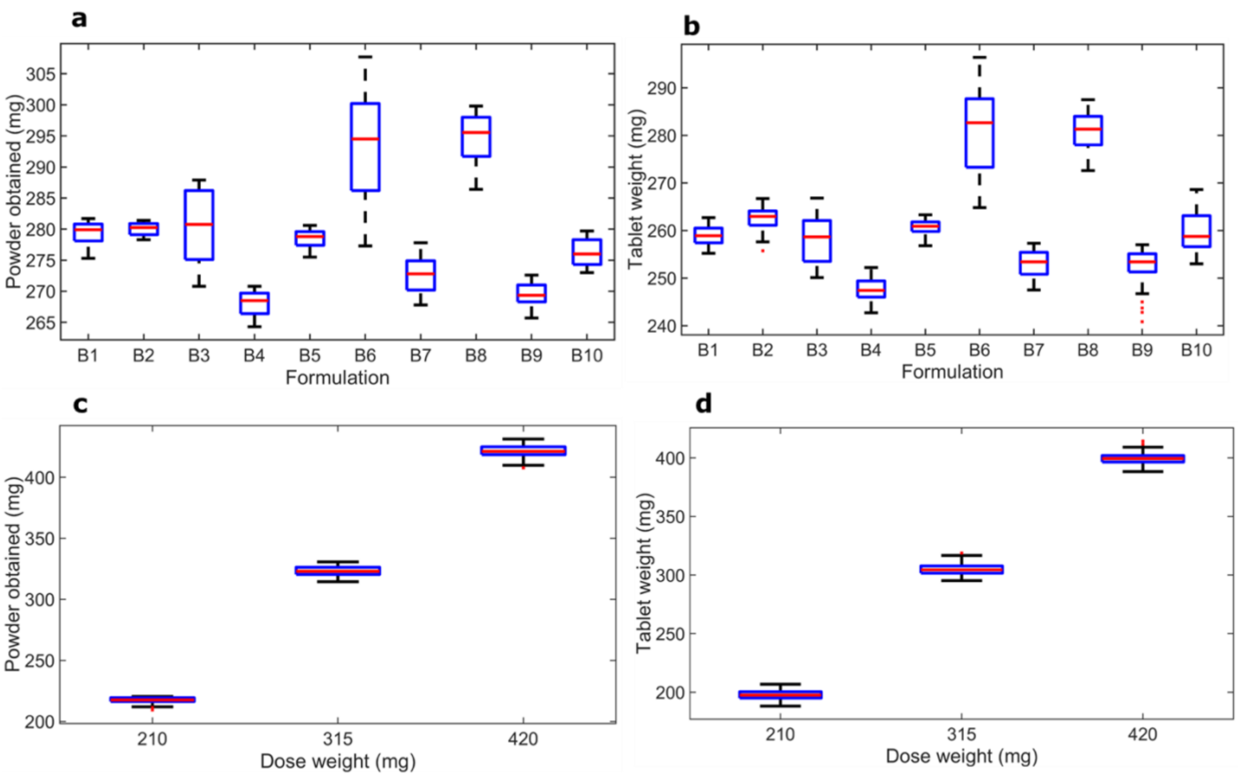

Supplementary Figure 23: Repeatability of powder obtained and tablet weight across formulations and dose weights. **a, b** shows powder obtained and tablet weight for different formulations and **c, d** shows different dose weights. Boxplots are based on 50 independent measurements per condition; the centre line denotes the median, boxes indicate the interquartile range (IQR), whiskers represent the data range excluding outliers, and points denote outliers. Source data are provided in the Source Data file.

3.1.9 Assessment of powder loss on content uniformity

Blends were prepared in a Turbula T2GE mixer to achieve formulations containing 5, 6, 7, 7.5, 8, 9, 10, 12.5, 15, 20 and 25 wt.% paracetamol (APAP), alongside 5% croscarmellose sodium (CCS), 1% magnesium stearate (MgSt), and 50:50 mixture of microcrystalline cellulose (MCC) and lactose (LAC) as the remaining excipients making up the %. Pre-lubrication mixing settings were 50 rpm for 10 min. Lubrication mixing settings were 50 rpm for 2.5 min.

Each blend was then subsampled in duplicate for high performance liquid chromatography (HPLC) analysis. The samples were analysed on a Thermo Fisher Vanquish Core system using an Agilent Zorbax Eclipse Plus C18 column (250 mm x 4.6 mm, 5 µm) maintained at 30°C. The mobile phase consisted of 30% acetonitrile and 70% water (v/v) delivered at a flow rate of 1.0 mL/min, and the total run time was set to 10 min. Each sample was dissolved, filtered, and diluted (working concentration: 0.15 mg mL^-1) appropriately. The injection volume set on the instrument method was 2 µL, the autosampler temperature was held at 5°C and the detection wavelength was set to 243 nm.

Following HPLC analysis, five subsamples of each blend were processed through the automated tablet development data factory. Each subsample was dosed in the 3D-printed TU, analysed by NIR, and then compacted into a tablet. Each tablet was then subsequently analysed again by NIRS using a similar 3D-printed unit which enabled accurate tablet placement.

The blend NIR data were pre-processed by wavelength trimming (1100-1450 nm), standard normal variate (SNV) transformation and smoothed via a Savitzky-Golay (SG) second derivative (polynomial order 2, window length 5). A partial least squares model (LV = 2) was produced to correlate the spectra with the reference HPLC measurements. K-fold cross-

validation, each fold containing five samples, was applied to evaluate predictive performance, see Supplementary Figure 24a.

The collected NIR spectra for both blends and tablets were subjected to wavelength trimming (1100-1450 nm), SNV transformation and smoothed via a SG second derivative (polynomial order 2, window length 5). Direct standardisation (DS) was then applied to correct blend spectra into the tablet spectral domain. For each fold in a 5-fold cross-validation set up, a subset of blend-tablet pairs was chosen as the training set, and the remaining pairs formed the test set. A least squares procedure was applied to the training set to solve:

$$\mathbf{X}_{\text{tablet}} = \mathbf{X}_{\text{blend}} \cdot \mathbf{A} \qquad \text{Supp. Equation (1)}$$

where, $\mathbf{X}_{\mathbf{blend}}$ and $\mathbf{X}_{\mathbf{tablet}}$ represent the blend and tablet spectra, respectively, and $\mathbf{A}$ is the transformation matrix that corrects the blend spectra to more closely resemble the tablet domain. The matrix $\mathbf{A}$ computed was then multiplied by the corresponding blend spectra in both training and test sets to produce DS-corrected blend spectra.

Following this, a partial least squares (PLS) regression model (LV =2) was fitted on each training fold using the DS corrected blend spectra and the average HPLC measurements from the original blends. The model was then applied to the DS corrected test spectra to predict content uniformity, yielding predictions which were compared to the HPLC measurements. After collection of all 5-fold predictions, every five subsamples corresponding to the same blend were grouped to measure the mean predicted values and standard deviations (Supplementary Figure 24b).

The DS corrected outputs ($R^2 = 0.92$, RMSE-CV = 2.01) indicate a robust correlation and low error, comparable to those obtained using the original blend NIR spectra ($R^2 = 0.97$, RMSE-CV = 0.84), as illustrated in Supplementary Figure 24. Despite unavoidable uncertainties introduced by sample preparation for HPLC, HPLC instrument variability, differences in NIR sampling of powders versus tablets, prediction error from PLS regression, and direct standardization (DS) errors, these findings strongly suggest that any powder losses during processing did not alter the overall composition of the blends. Material was lost in bulk rather than selectively, the ratio of paracetamol to excipients remained stable throughout the process.

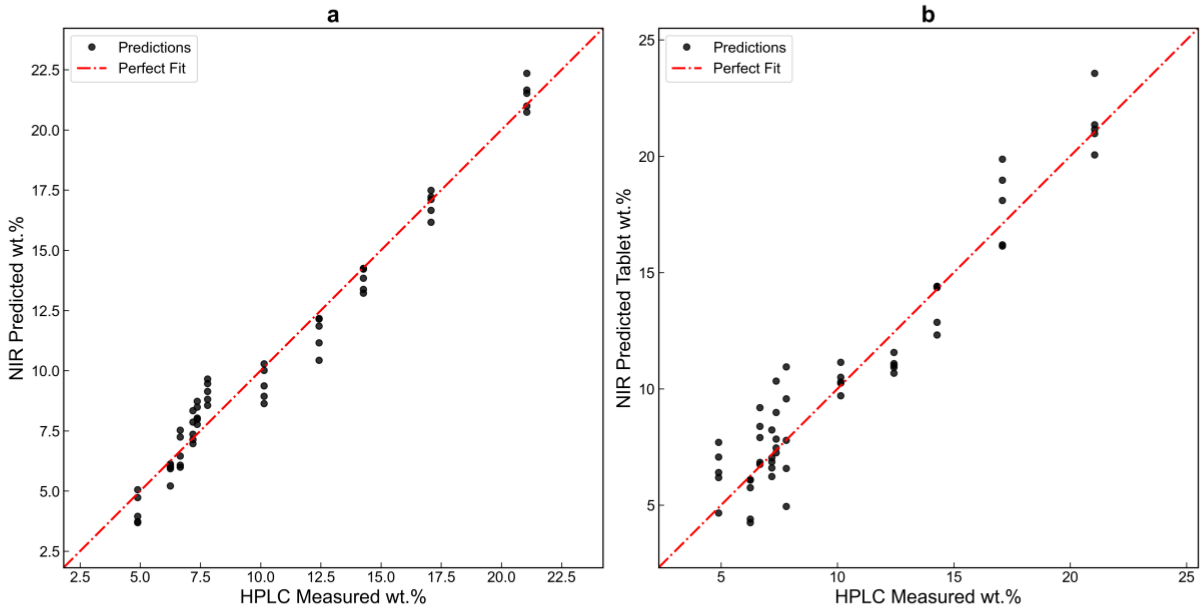

Supplementary Figure 24: Depicted are the NIR PLS-CV regression results using **a** the original blend spectra and **b** the DS-corrected blend spectra targeting the tablet domain. Individual NIR predicted vs. HPLC measured paracetamol content (wt.%) values are shown for all blend samples processed through the automated tablet development data factory. The red dashed line indicates the perfect one-to-one relationship between NIR predicted and HPLC measured values. Source data are provided as a Source Data file.

3.2 Experimental agent

3.2.1 Background of Bayesian optimisation

Bayesian optimisation (BO) is a framework for optimising expensive-to-evaluate functions, particularly in high-dimensional or non-convex spaces where traditional optimisation methods may fail [12]. BO leverages a probabilistic surrogate model, often a Gaussian Process (GP), to model the objective function $\ell(\underline{X})$, where $\underline{X}$ is the state vector of input parameters. Given this prohibitively expensive objective function, the uncertainty of the objective $\ell(\cdot)$ across not-yet-evaluated input points is modelled as a probability distribution. BO models $\ell(\cdot)$ as a GP, which can be evaluated relatively cheaply and often with reasonable accuracy [13]. At each iteration the GP model is used to select the most promising candidate $\underline{X}^*$ for evaluation. The costly function ℓ is then only evaluated at $\ell(\underline{X}^*)$ in this iteration. Subsequently, the GP updates its posterior belief $\tilde{\ell}(\cdot)$ with the new data pair $(\underline{X}^*, \ell(\underline{X}^*))$, and that pair is added to the known experiment set $\mathcal{D}_n = \left\{\left(\underline{X}_i, \ell(\underline{X}_i)\right)\right\}_{i=1}^{n}$. This iteration can be repeated to iterate to an optimum. The critical step is the selection of the candidate point $\underline{X}^*$, which is performed via an acquisition function that enables active learning of the objective $\ell(\cdot)$ [14]. The acquisition function $\alpha(\underline{X})$ guides the selection of the next evaluation point $\underline{X}_{n+1}$ by quantifying the utility of evaluating $\ell(\underline{X})$ at a given point, typically formulated as $\underline{X}_{n+1} = \arg\max_{\underline{X}} \alpha(\underline{X} | \mathcal{D}_n)$. This iterative process continues until convergence criteria are met. Common acquisition functions include the expected improvement (EI), upper confidence bound (UCB), and probability of improvement (PI).[15] In this study, EI is used the acquisition function due to its proven efficiency in balancing between the exploration and exploitation[16]

3.2.2 Digital integration of optimisation frameworks with tableting data factory

Both optimisation frameworks, PIBO and MOBO, were digitally integrated with the tableting data factory through a local call mechanism between LabVIEW and Python scripts. LabVIEW

triggered the Python scripts to execute the optimisation routines, ensuring seamless communication and data flow between the control system and the optimisation algorithms. Each experiment was repeated three times for enhanced consistency, with the average value from three tablets used in the optimisation workflow.

3.2.3 Multi-output Bayesian optimisation (MOBO) for rapid scale-up assessment

During scale-up, process parameters can behave differently due to increased speeds and forces, requiring careful consideration of intercorrelation between multiple process parameters.[2] A scale-up, multi-output Bayesian framework was designed to minimise elastic recovery while achieving target porosity and tensile strength by optimising key process parameters such as main compression pressure, precompression pressure, and dwell time. Minimising elastic recovery is critical, as it is closely associated with tablet defects such as lamination, capping, and air entrapment, particularly when scaling up from compaction simulators to rotary tablet presses.[17, 18]

For the MOBO, a design of experiments (DoE) using the Latin hypercube sampling (LHS) method was initially employed to generate a diverse set of experimental conditions to pre-train the process models.[19] LHS is used due to its space-filling features to generate training datasets that are evenly distributed over the design space to ensure good coverage. This was followed by a classic black-box optimisation approach to iteratively refine the decision parameters space. Three independent GP models were trained during MOBO to individually predict elastic recovery, porosity, and tensile strength based on the input parameters. The target was set to minimise the elastic recovery while meeting the porosity ($\varepsilon \geq 0.15$) and tensile strength ($\sigma \geq$ 2 MPa) constraints. The LHS was run for 15 experiments followed by 25 iterations of Bayesian optimisation. The optimisation process was terminated after the predetermined number of experiments.

4 Demonstration of platform workflow

4.1 Formulations

Four distinct quinary blends, exhibiting varying concentrations of paracetamol at 16%, 18%, 20% and 22%, and 20 wt.%, respectively, were formulated as listed in Supplementary Table 9. All the blends were prepared using a laboratory powder blender (PharmaTech Multiblend MB015). The blending process was performed at a blender speed of 20 rpm and an agitator speed of 200 rpm over a duration of 20 min to ensure homogeneity. In addition to the formulation process, all blends were lubricated with 1 wt.% of magnesium stearate, a procedure implemented to improve the flow properties of the blends. This lubrication step was executed by allowing the blend to mix for an additional 5 min, thus ensuring a uniform distribution of the lubricant without overlubricating the mixture. In our study, we also prepared five quinary blends using different APIs, as illustrated in Supplementary Table 9, each planned to include a fixed concentration of the API (20 wt.%), croscarmellose sodium (3.5 wt.%) and magnesium stearate (1%). These blends were designed to have varied filler ratios to broaden the knowledge space. The filler combinations utilised included different grades of microcrystalline cellulose and lactose. The combination and concentrations of fillers were optimized using models. Consistency was maintained in the formulation process across the blends, employing the same laboratory powder blender (PharmaTech Multiblend MB015) with fixed settings of a 20-rpm blender speed and a 200-rpm agitator speed, operating over a duration of 20 min. A detailed overview of the precise formulations and the corresponding concentrations (wt.%) of each filler in each blend prepared are illustrated in Supplementary Table 9.

Supplementary Table 9: Composition of the tablet blends predicted by the digital formulator and tested by the tableting data factory. The data in the table represents the percentage weight by weight (wt.%) of each ingredient in the formulation. Each entry in the table specifies the unique identifier of API and excipients followed by the concentration.

**API (wt.%)**	**Disintegrant (wt.%)**	**Filler/Binder 1 (wt.%)**	**Filler/Binder 2 (wt.%)**	**Lubricant (wt.%)**
**SP (16%)**	CCS (3.5%)	LAC1 (30.4%)	MCC2 (49.1%)	MgSt (1%)
**SP (18%)**	CCS (3.5%)	LAC1 (23%)	MCC2 (54.5%)	MgSt (1%)
**SP (20%)**	CCS (3.5%)	LAC1 (2.7%)	MCC2 (72.8%)	MgSt (1%)
**SP (22%)**	CCS (3.5%)	-	MCC2 (73.5%)	MgSt (1%)
**AS (20%)**	CCS (3.5%)	LAC1 (27.7%)	MCC2 (47.8%)	MgSt (1%)
**DM (20%)**	CCS (3.5%)	MCC1 (57.3%)	MCC2 (18.2%)	MgSt (1%)
**GR (20%)**	CCS (3.5%)	LAC1 (41.3%)	MCC3 (34.2%)	MgSt (1%)
**IM (20%)**	CCS (3.5%)	LAC1 (57.3%)	MCC2 (18.2%)	MgSt (1%)
**MH (20%)**	CCS (3.5%)	LAC1 (30.5%)	MCC2 (45%)	MgSt (1%)

4.2 Digital formulator

Supplementary Table 10: List of decision parameters and their values/ranges in the formulation optimisation cases.

Decision parameter	Value/Range
API	SP, AS, DM, GR, IM, MH
API mass fraction	0.16, 0.18, 0.2, 0.22
Excipient 1	Choice from MCC (3 grades), LAC (2 grades), MAN (1 grade)
Excipient 1 mass fraction	[0 – 1]
Excipient 2	Choice from MCC (3 grades), LAC (2 grades), MAN (1 grade)
Excipient 2 mass fraction	[0 – 1]
Lubricant	MgSt
Lubricant mass fraction	0.035
Disintegrant	CCS
Disintegrant mass fraction	0.01
Compression Pressure (MPa)	[70 – 450]

4.2.1 Flowability model performance

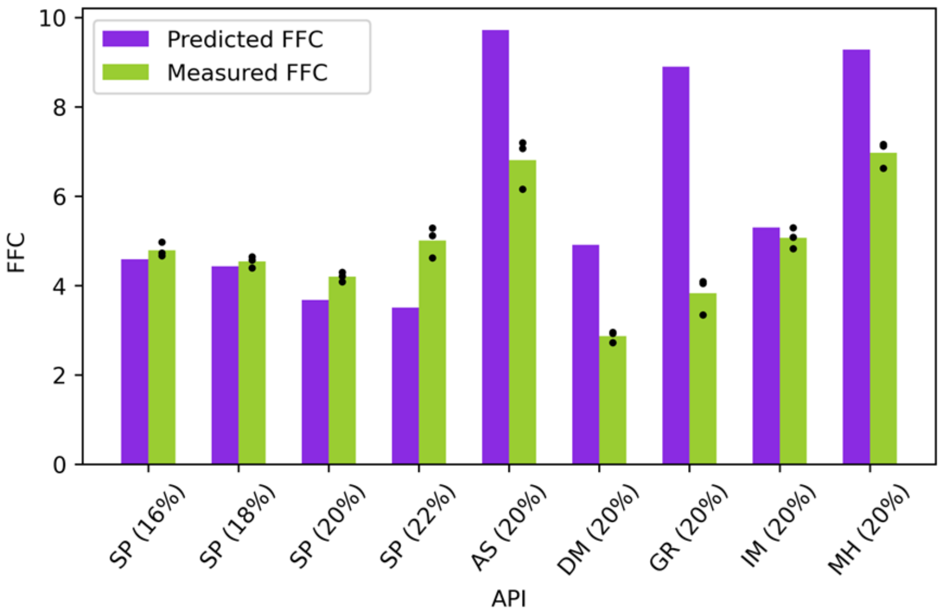

Supplementary Figure 25: Validation of FFC values against the measured data (over three repeats, individual data points shown in black) at consolidation pressure of 1.6 KPa. Source data are provided as a Source Data file.

4.2.2 Parameterisation of distributions for API particle size assessment on blend and tablet properties

Unimodal log-normal distribution:

The log-normal probability density function (PDF) describes a random variable X whose natural logarithm follows a normal distribution:

$$\ln\, X \sim \mathcal{N}(\mu, \sigma^2) \qquad \text{Supp. Equation (2)}$$

The PDF of X is derived via transformation of variables:

$$f_X(x) = \frac{d}{dx} F_X(x) = \frac{d}{dx} P(X \leq x) \qquad \text{Supp. Equation (3)}$$

Since $\ln\, X \sim \mathcal{N}(\mu, \sigma^2)$:

$$P(X \leq x) = P(\ln\, X \leq \ln\, x) = \Phi\left(\frac{\ln\, x - \mu}{\sigma}\right) \qquad \text{Supp. Equation (4)}$$

where Φ is the cumulative distribution function (CDF) of the standard normal distribution. Differentiating with respect to x:

$$f_X(x) = \frac{d}{dx} \Phi\left(\frac{\ln\, x - \mu}{\sigma}\right) = \phi\left(\frac{\ln\, x - \mu}{\sigma}\right) \cdot \frac{1}{\sigma x} \qquad \text{Supp. Equation (5)}$$

where $\phi(z) = \frac{1}{\sqrt{2\pi}} e^{-z^2/2}$ is the standard normal PDF. This yields:

$$f_X(x) = \frac{1}{x\sigma\sqrt{2\pi}} \exp\left(-\frac{(\ln\, x - \mu)^2}{2\sigma^2}\right) \qquad \text{Supp. Equation (6)}$$

To parameterise the PDF above based D_{50} and span, we define D_{50} as the diameter where $F_X(D_{50}) = 0.5$:

$$\ln\,(D_{50}) = \mu \;\Rightarrow\; \mu = \ln\,(D_{50}) \qquad \text{Supp. Equation (7)}$$

Next, span is defined as $\frac{D_{90}-D_{10}}{D_{50}}$, where D_{10} is the 10th percentile ($P(X \leq D_{90}) = 0.10$) and D_{90} is the 90th percentile ($P(X \leq D_{90}) = 0.90$). Using the quantile function of $\ln(X)$:

$$D_{10} = \exp\left(\mu + \sigma \cdot \Phi^{-1}(0.10)\right) = \exp\left(\mu - 1.2816\sigma\right) \quad \text{Supp. Equation (8)}$$

$$D_{90} = \exp\left(\mu + \sigma \cdot \Phi^{-1}(0.90)\right) = \exp\left(\mu + 1.2816\sigma\right) \quad \text{Supp. Equation (9)}$$

Substituting into the span definition:

$$\text{Span} = \frac{\exp\left(\mu + 1.2816\sigma\right) - \exp\left(\mu - 1.2816\sigma\right)}{\exp\left(\mu\right)} \quad \text{Supp. Equation (10)}$$

$$= \exp\left(1.2816\sigma\right) - \exp\left(-1.2816\sigma\right)$$

Supp. Equation (10 is solved numerically for σ given a target span, while $\mu = \ln\left(D_{50}\right)$ is directly determined from D_{50}.

Bimodal distribution: The bimodal distribution combines two log-normal distributions as outlined above:

$$f_{\text{bimodal}}(x) = \alpha \cdot f_{X_1}(x) + (1-\alpha) \cdot f_{X_2}(x) \quad \text{Supp. Equation (11)}$$

where $f_{X_i}(x)$ is the PDF of a log-normal distribution with parameters (μ_i, σ_i), and α is the mixture weight, which is numerically adjusted to achieve the desired median and span values for each component of the mixture ($i = 2$ in this work). Supplementary Table 11 and Supplementary Table 12, respectively, summarise the user-defined parameters to generate unimodal (UMD) and bimodal (BMD) distributions.

Supplementary Table 11: Summary of parameters used to generate unimodal distributions (UMDs). P_{20} denotes the percentage of particles below 20 μm, a description of fraction of fine particles in each generated distribution.

**Distribution ID**	$\boldsymbol{D_{50}}$ **(μm)**	**Span**	$\boldsymbol{P_{20}}$ **(%)**
UMD1	10	1.15	95.92
UMD2	10	1.7	89.15
UMD3	80	1.4	0.4275
UMD4	100	1.15	0.0125
UMD5	100	1.6	0.3143
UMD6	150	1.3	0.0018
UMD7	200	1.15	0.0001
UMD8	250	1.5	0.0002

Supplementary Table 12: Summary of parameters used to generate bimodal distributions (BMDs). P_{20} denotes the percentage of particles below 20 μm, a description of fraction of fine particles in each generated distribution. The subscript 1 and 2 of the D_{50} and $Span$ refers to the log-normal distributions f_{x_1} and f_{x_2}, respectively, that are used to generate the bimodal size distribution. α denotes the mixture weight.

**Distribution ID**	D_{50_1} **(μm)**	$Span_1$	D_{50_2} **(μm)**	$Span_2$	α	P_{20} **(%)**
BMD1	10	1.15	300	1.15	0.4	38.37
BMD2	10	1.4	300	1.15	0.35	32.47
BMD3	10	1.7	300	1.15	0.3	26.77
BMD4	100	1.15	300	1.15	0.45	0.0056
BMD5	100	1.4	300	1.15	0.5	0.0538
BMD6	100	1.7	300	1.15	0.35	0.1653
BMD7	150	1.15	300	1.15	0.55	0.0001
BMD8	150	1.4	300	1.15	0.5	0.0028

Supplementary Table 13: Summary of simulated blend FFC values (at 1.6 KPa) using the generated API distributions at varying drug loadings.

	**Drug loading (wt.%)**					
**Distribution ID**	**0%**	**20%**	**40%**	**60%**	**80%**	**100%**
UMD1	6.52	3.43	2.75	2.07	1.42	1.18
UMD2	6.52	3.86	2.89	2.21	1.62	1.38
UMD3	6.52	4.04	3.39	2.83	2.24	2.01
UMD4	6.52	4.43	4.02	3.19	2.68	2.21
UMD5	6.52	5.13	4.69	4.37	3.84	3.43
UMD6	6.52	5.60	5.01	4.59	4.30	3.79
UMD7	6.52	6.64	7.03	7.46	7.94	8.24
UMD8	6.52	5.86	5.21	4.83	4.41	4.11
BMD1	6.52	3.94	3.70	3.19	2.82	2.51
BMD2	6.52	4.53	3.81	3.53	3.15	2.87
BMD3	6.52	4.65	4.02	3.67	3.41	3.10
BMD4	6.52	5.09	4.59	4.18	3.92	3.68
BMD5	6.52	5.36	4.57	4.41	3.99	3.65
BMD6	6.52	5.40	5.10	4.81	4.46	3.97
BMD7	6.52	6.08	6.51	6.80	7.08	7.38
BMD8	6.52	6.74	6.96	7.43	7.86	8.02

Supplementary Table 14: Summary of simulated tablet tensile strength values at 0.15 porosity using the generated API distributions at varying drug loadings.

	**Drug loading (wt.%)**					
**Distribution ID**	**0%**	**20%**	**40%**	**60%**	**80%**	**100%**
UMD1	2.21	2.20	1.75	1.35	1.00	0.70
UMD2	2.21	2.18	1.73	1.33	0.98	0.69
UMD3	2.21	2.18	1.74	1.34	0.99	0.69
UMD4	2.21	2.19	1.74	1.34	0.99	0.70
UMD5	2.21	2.17	1.73	1.32	0.98	0.68
UMD6	2.21	2.17	1.73	1.32	0.98	0.69
UMD7	2.21	2.16	1.72	1.32	0.98	0.69
UMD8	2.21	2.14	1.70	1.30	0.96	0.67
BMD1	2.21	2.20	1.90	1.45	0.95	0.60
BMD2	2.21	2.19	1.89	1.44	0.95	0.59
BMD3	2.21	2.19	1.88	1.43	0.94	0.58
BMD4	2.21	2.19	1.89	1.44	0.94	0.60
BMD5	2.21	2.19	1.88	1.43	0.94	0.59
BMD6	2.21	2.18	1.87	1.42	0.93	0.58
BMD7	2.21	2.19	1.88	1.43	0.94	0.59
BMD8	2.21	2.18	1.87	1.42	0.93	0.58

4.2.3 Sensitivity analysis of optimal formulations

To assess the robustness of the solutions generated by the digital formulator and quantify the trade-offs between conflicting objectives, a sensitivity analysis was conducted on the excipient composition. For each optimised formulation, the mass fraction of the primary filler (e.g., LAC or MCC) was systematically varied across the feasible design space while maintaining fixed drug (API) loading. This section illustrates the predicted trajectories for two critical quality attributes: Flow function coefficient (FFC) at 1.6 kPa and tensile strength at a reference porosity of 0.15. The model-based epistemic uncertainty is encoded within the visualisations, where the colour gradient represents the standard deviation of the ensemble predictions for both responses.

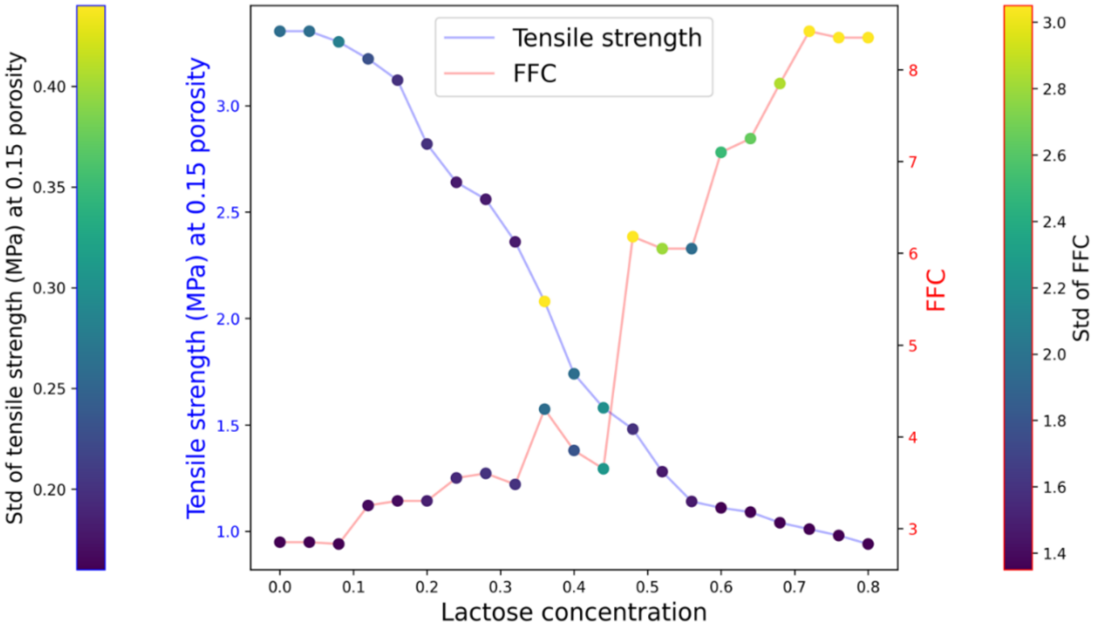

Supplementary Figure 26: Variation of predicted tensile strength at 0.15 porosity and FFC (at 1.6 KPa) with excipient concentrations in optimal formulation 1 (SP 16 wt.%). Source data are provided as a Source Data file.

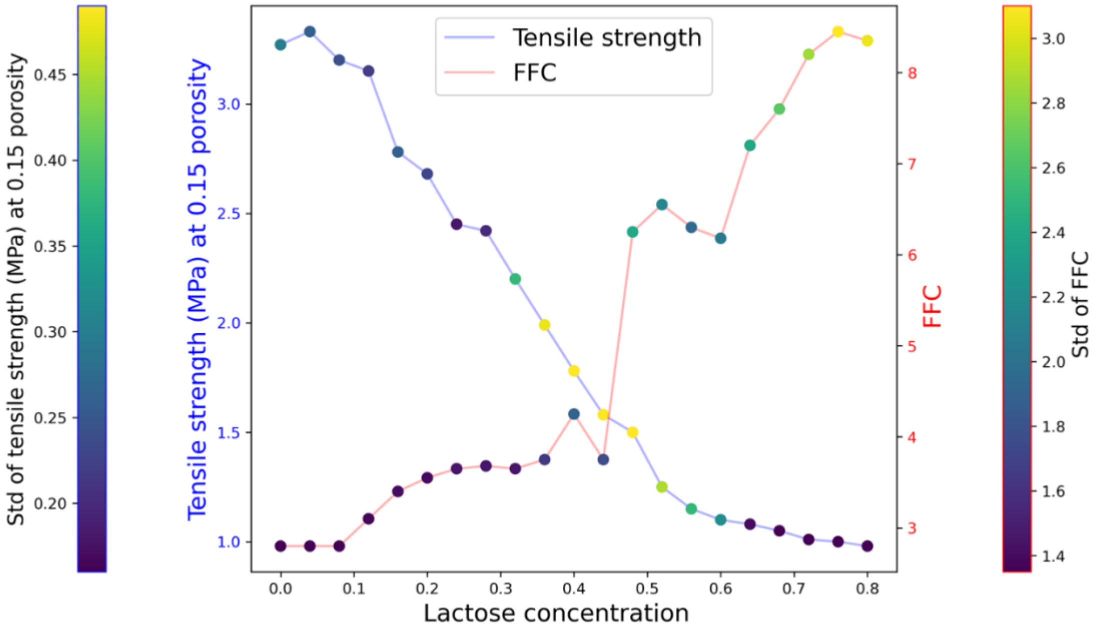

Supplementary Figure 27: Variation of predicted tensile strength at 0.15 porosity and FFC (at 1.6 KPa) with excipient concentrations in optimal formulation 2 (SP 18 wt.%). Source data are provided as a Source Data file.

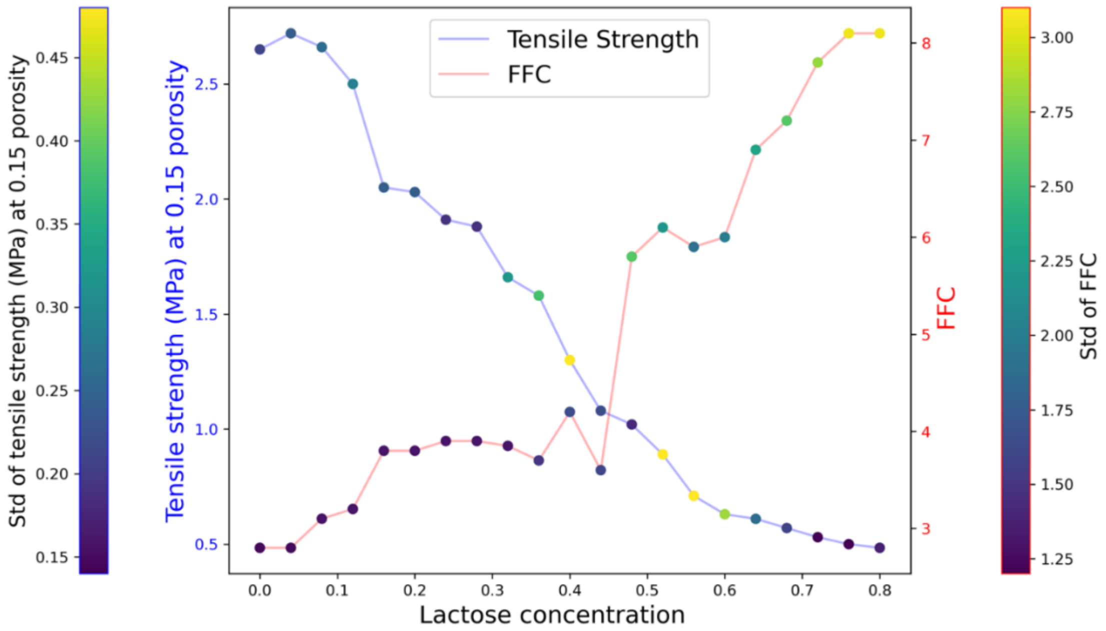

Supplementary Figure 28: Variation of predicted tensile strength at 0.15 porosity and FFC (at 1.6 KPa) with excipient concentrations in optimal formulation 3 (SP 20 wt.%). Source data are provided as a Source Data file.

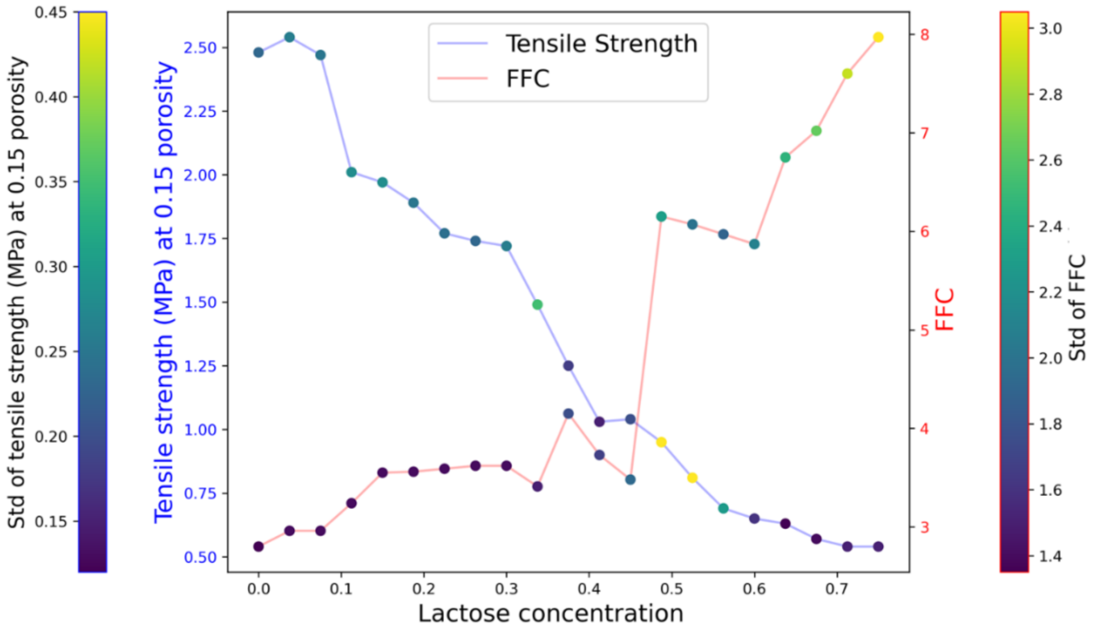

Supplementary Figure 29: Variation of predicted tensile strength at 0.15 porosity and FFC (at 1.6 KPa) with excipient concentrations in optimal formulation 4 (SP 22 wt.%). Source data are provided as a Source Data file.

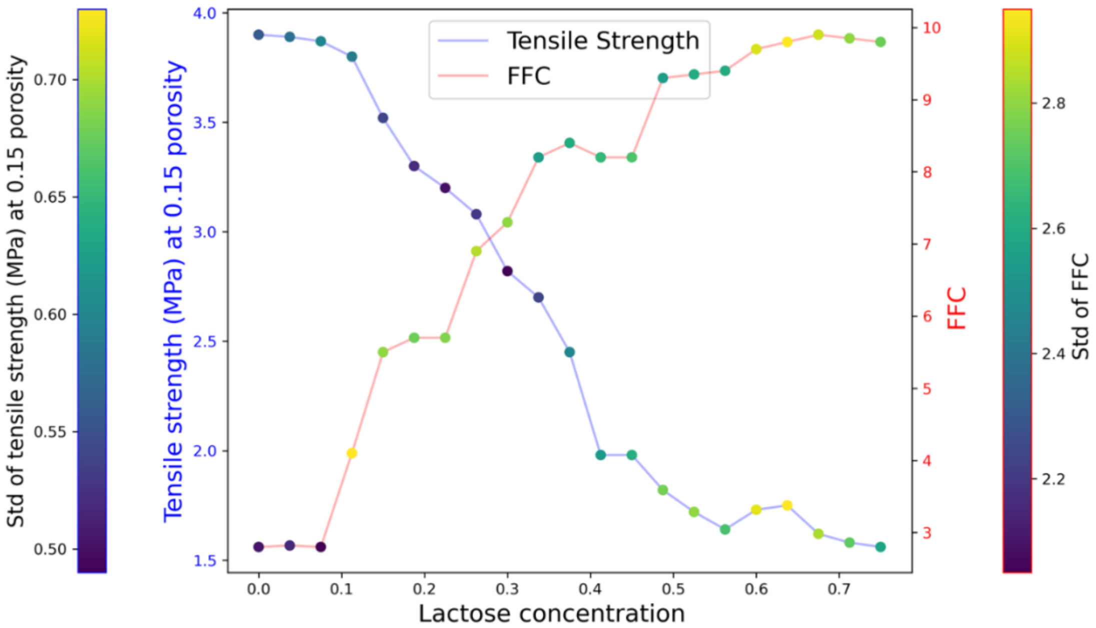

Supplementary Figure 30: Variation of predicted tensile strength at 0.15 porosity and FFC (at 1.6 KPa) with excipient concentrations in optimal formulation 5 (AS 20 wt.%). Source data are provided as a Source Data file.

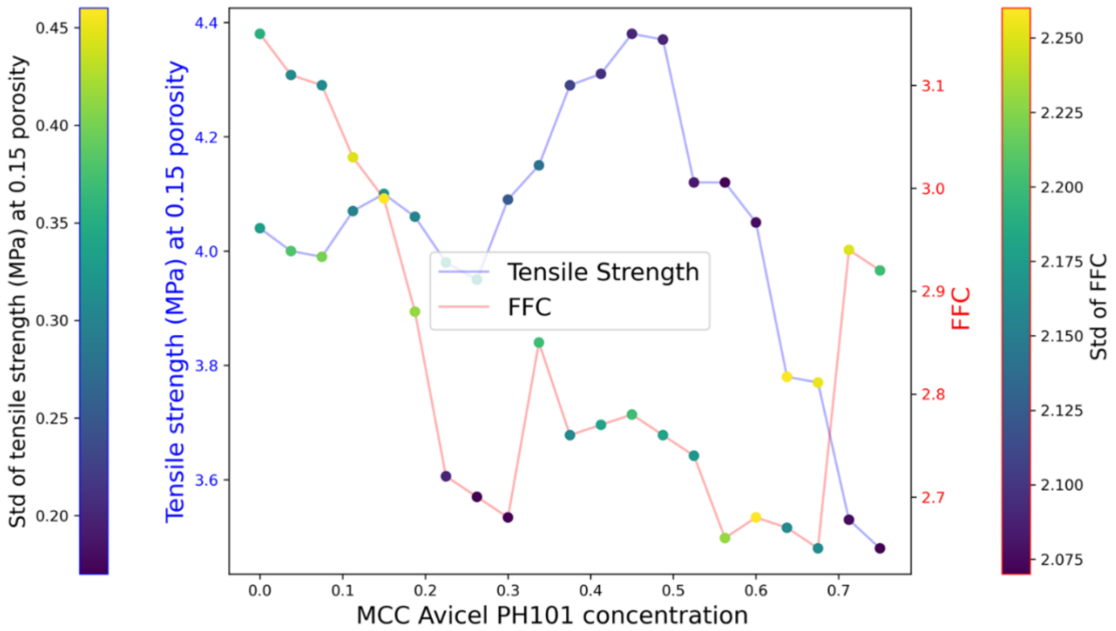

Supplementary Figure 31: Variation of predicted tensile strength at 0.15 porosity and FFC (at 1.6 KPa) with excipient concentrations in optimal formulation 6 (DM 20 wt.%). Source data are provided as a Source Data file.

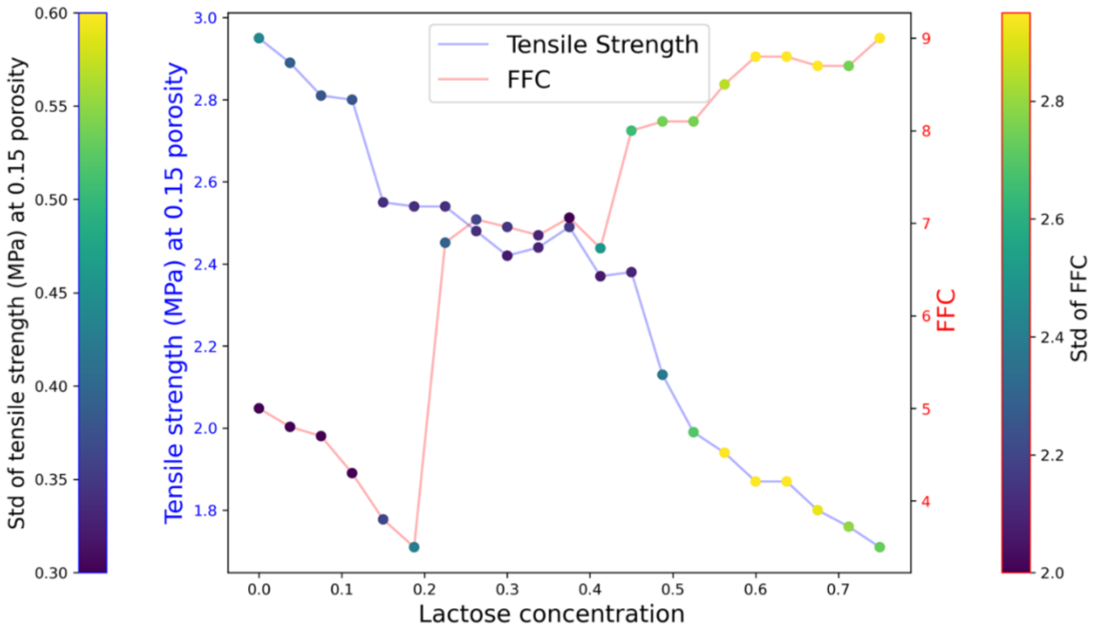

Supplementary Figure 32: Variation of predicted tensile strength at 0.15 porosity and FFC (at 1.6 KPa) with excipient concentrations in optimal formulation 7 (GR 20 wt.%). Source data are provided as a Source Data file.

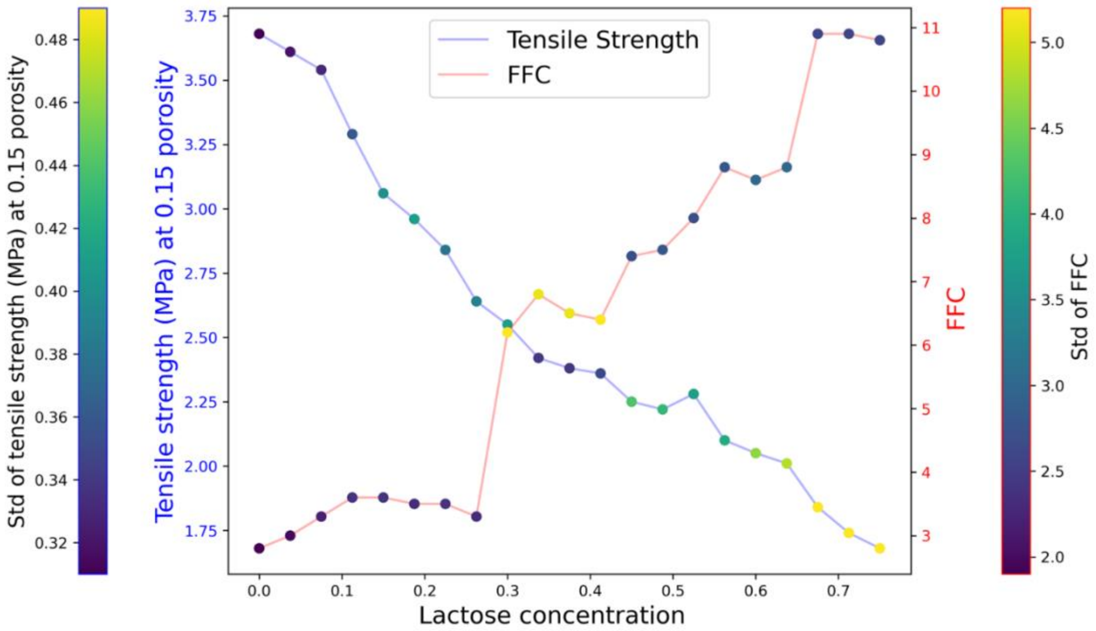

Supplementary Figure 33: Variation of predicted tensile strength at 0.15 porosity and FFC (at 1.6 KPa) with excipient concentrations in optimal formulation 8 (IM 20 wt.%). Source data are provided as a Source Data file.

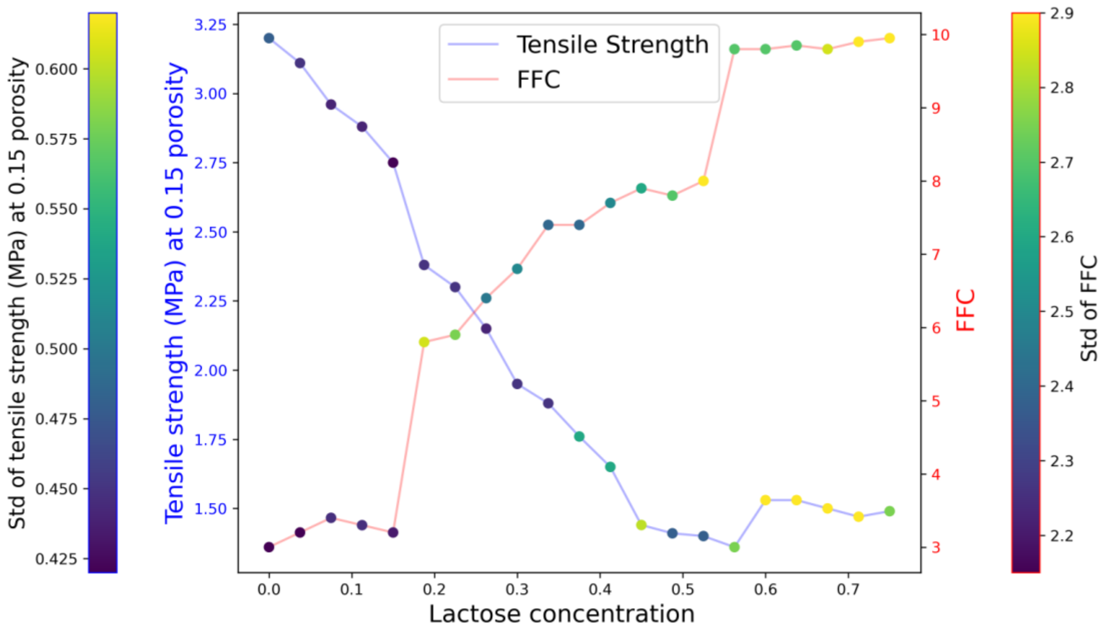

Supplementary Figure 34: Variation of predicted tensile strength at 0.15 porosity and FFC (at 1.6 KPa) with excipient concentrations in optimal formulation 9 (MH 20 wt.%). Source data are provided as a Source Data file.

4.3 Tableting data factory

4.3.1 Near-infrared (NIR) spectroscopy

However, raw NIR spectra often suffer from baseline shifts, scattering effects, and noise, necessitating robust pre-processing and dimensionality reduction techniques to ensure reliable analysis. This study incorporates trimming, standard normal variate (SNV) transformation, and Savitzky-Golay (SG) filtering for pre-processing. Principal component analysis (PCA) is used for dimensionality reduction, while Hotelling's T^2 analysis is employed for outlier detection and quality assessment of the spectral dataset.

NIR spectra pre-processing

Wavelength reduction: The raw spectra were trimmed to the range 1050-1450 nm, where relevant chemical information to paracetamol was present.

Standard normal variate (SNV): Each spectrum ($\mathbf{X}_{\text{blend}}$) was normalised to minimise scattering effects and baseline shifts. The transformation was applied as:

$$\mathbf{X}'_{\text{blend}} = \frac{\mathbf{X}_{\text{blend}} - \mathbf{X}_{\text{blend,mean}}}{\mathbf{X}_{\text{blend,std.dev}}} \quad \text{Supp. Equation (12)}$$

Savitzky-Golay (SG) smoothing and derivation: SG filter was applied to compute the first derivative of each spectrum, enhancing spectral features and reducing noise. Parameters used include: Window: 8; Polynomial Order: 2; Derivative Order: 1.

Principal component analysis (PCA)

PCA was applied on the pre-processed spectra to reduce dimensionality and extract key patterns. The PCA transformation is described by:

$$\mathbf{T} = \mathbf{X}'_{\text{blend}} \cdot \mathbf{P} \quad \text{Supp. Equation (13)}$$

where **T** represents the scores, **P** represents the loadings, and $\mathbf{X}'_{\text{blend}}$ is the pre-processed spectra matrix. The first three principal components were retained, capturing 95% of the total variance in the spectral dataset.

Hotelling's T^2 Analysis

Hotelling's T^2 statistic was used to evaluate the multivariate distance of each spectrum from the PCA model centre. The T^2 value for each sample was calculated as:

$$T_i^2 = \sum_{j=1}^{k} \left(\frac{t_{ij}^2}{\lambda_j} \right) \quad \text{Supp. Equation (14)}$$

where, t_{ij} is the score of the ith spectrum on the jth PC, λ_j is the variance of the jth PC, and k is the number of retained components (here, $k = 3$). The control limit was determined based on chi-squared (χ^2) distribution at a 99% confidence level:

$$Control\ Limit = \chi^2_{0.99,k} \quad \text{Supp. Equation (15)}$$

For $k = 3$, the control limit was 11.34. Spectral outliers were identified where $T_i^2 > 11.34$. Detected outliers occurred at iterations 3 and 36, see Supplementary Figure 36.

Integration of NIR with tableting data factory

NIR data collection and pre-processing were digitally integrated into the experimental workflow, enabling real-time spectral analysis. Spectra from 100 consecutive samples were recorded and processed, with visualisations generated for both raw and pre-processed data, see Supplementary Figure 35. This allowed for the efficient detection of potential anomalies and enhanced confidence in the experimental outcomes.

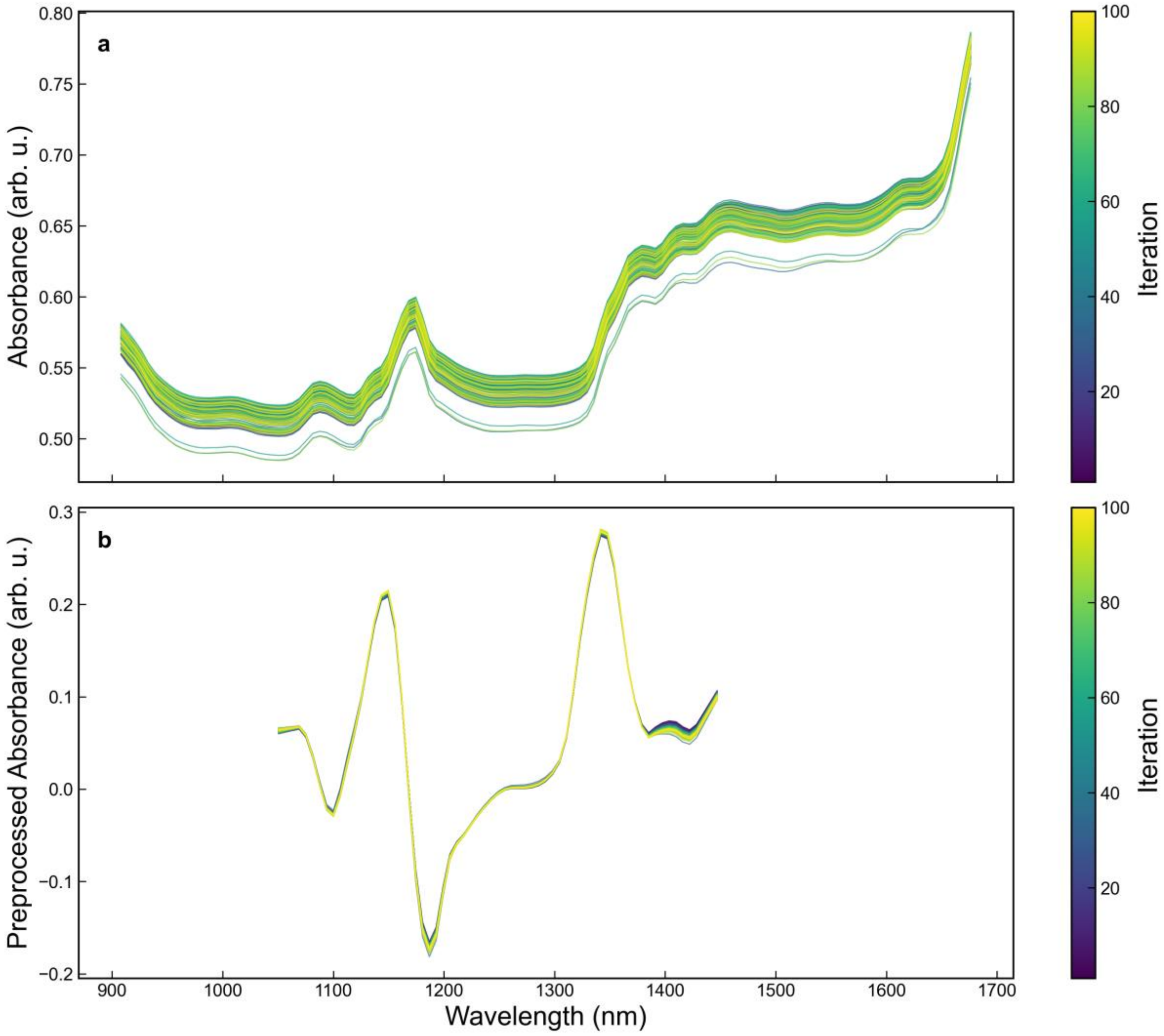

Supplementary Figure 35: **a** Raw near-infrared (NIR) spectra collected from 100 consecutive samples, plotted as absorbance (arb. u.) across the wavelength range of 908–1676 nm. Each spectrum is colour-coded by its iteration number, with earlier samples shown in purple and later samples transitioning to yellow. **b** Pre-processed spectra of the same samples, following trimming to 1050–1450 nm wavelength range, standard normal variate (SNV) correction, and Savitzky-Golay smoothing and derivation. The colour coding corresponds to the same iteration numbers as in **a**. Source data are provided as a Source Data file.

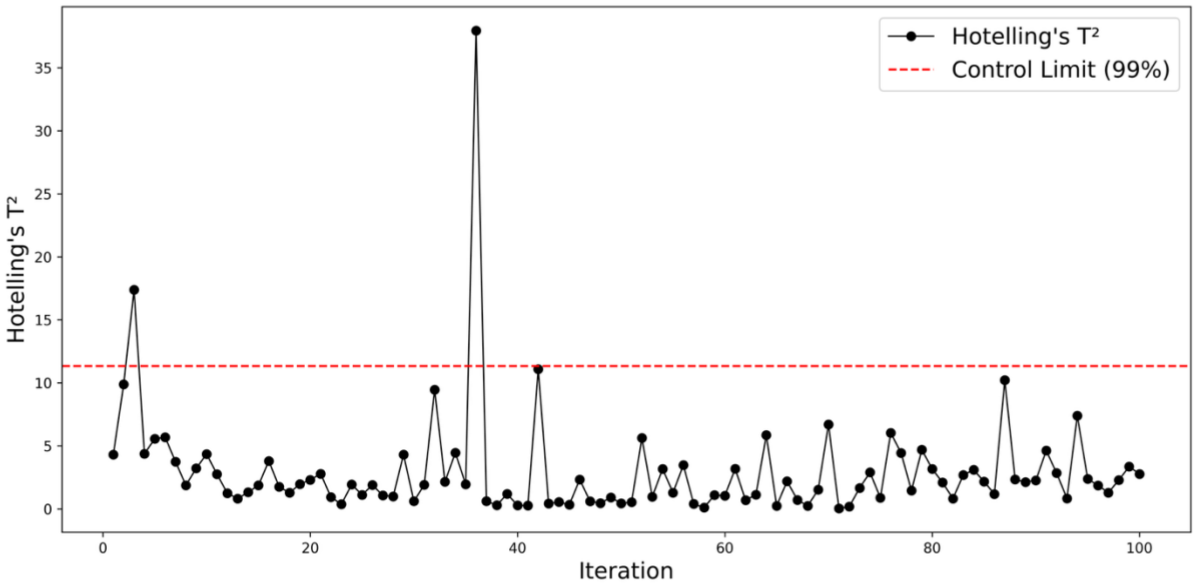

Supplementary Figure 36: Hotelling's T^2 chart for 100 consecutive samples, calculated using the first three principal components from the pre-processed NIR spectra. Each data point represents the T^2 statistic for an individual sample, quantifying its multivariate distance from the PCA model centre. The red dashed line indicates the control limit at the 99% confidence level ($T^2 > 11.34$). Source data are provided as a Source Data file.

4.3.2 Physics-informed Bayesian optimisation (PIBO)

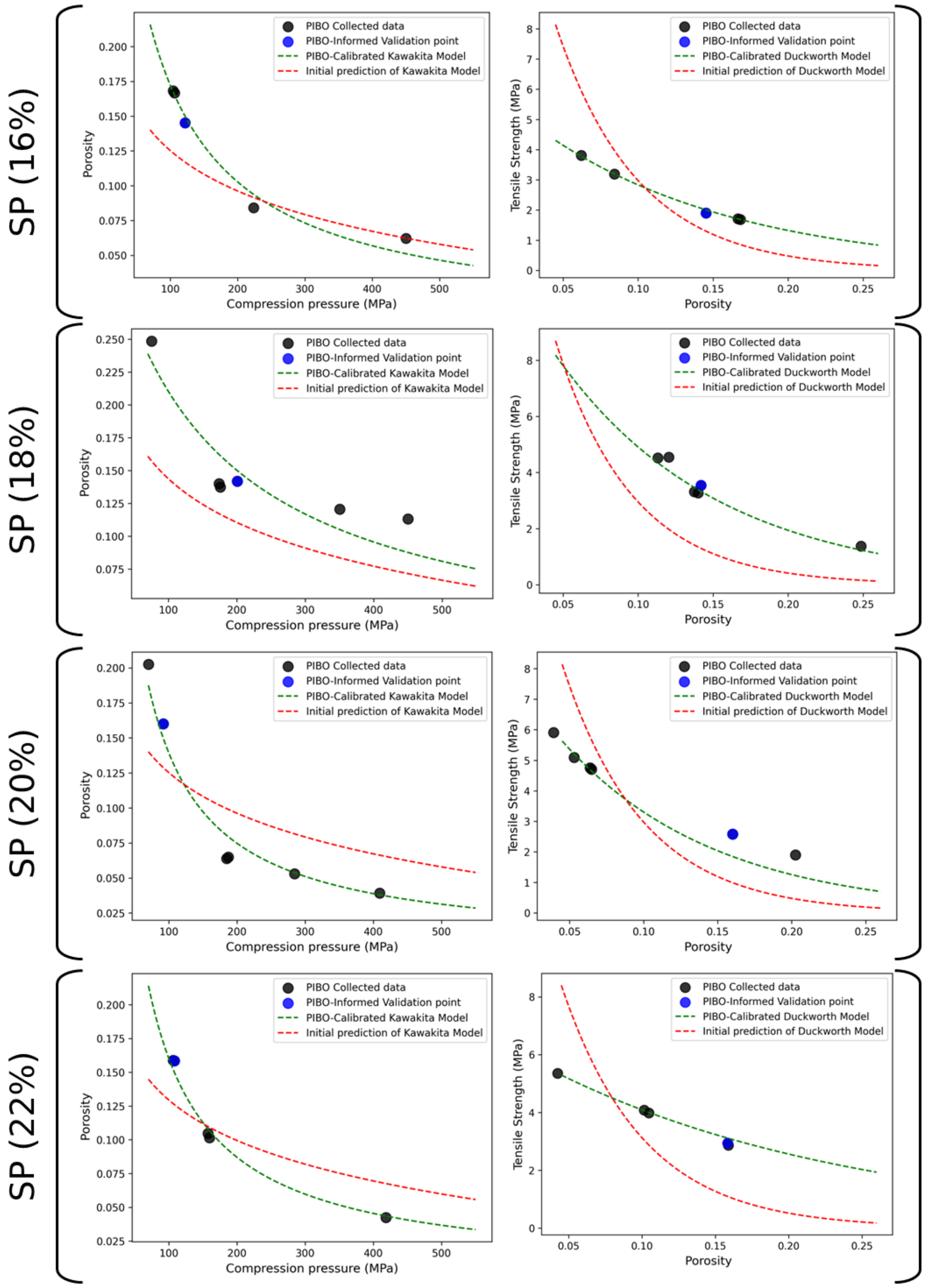

Supplementary Figure 37: Initial and calibrated prediction of compressibility and compactability profiles before (using the system of models) and after the calibration with PIBO. Source data are provided as a Source Data file.

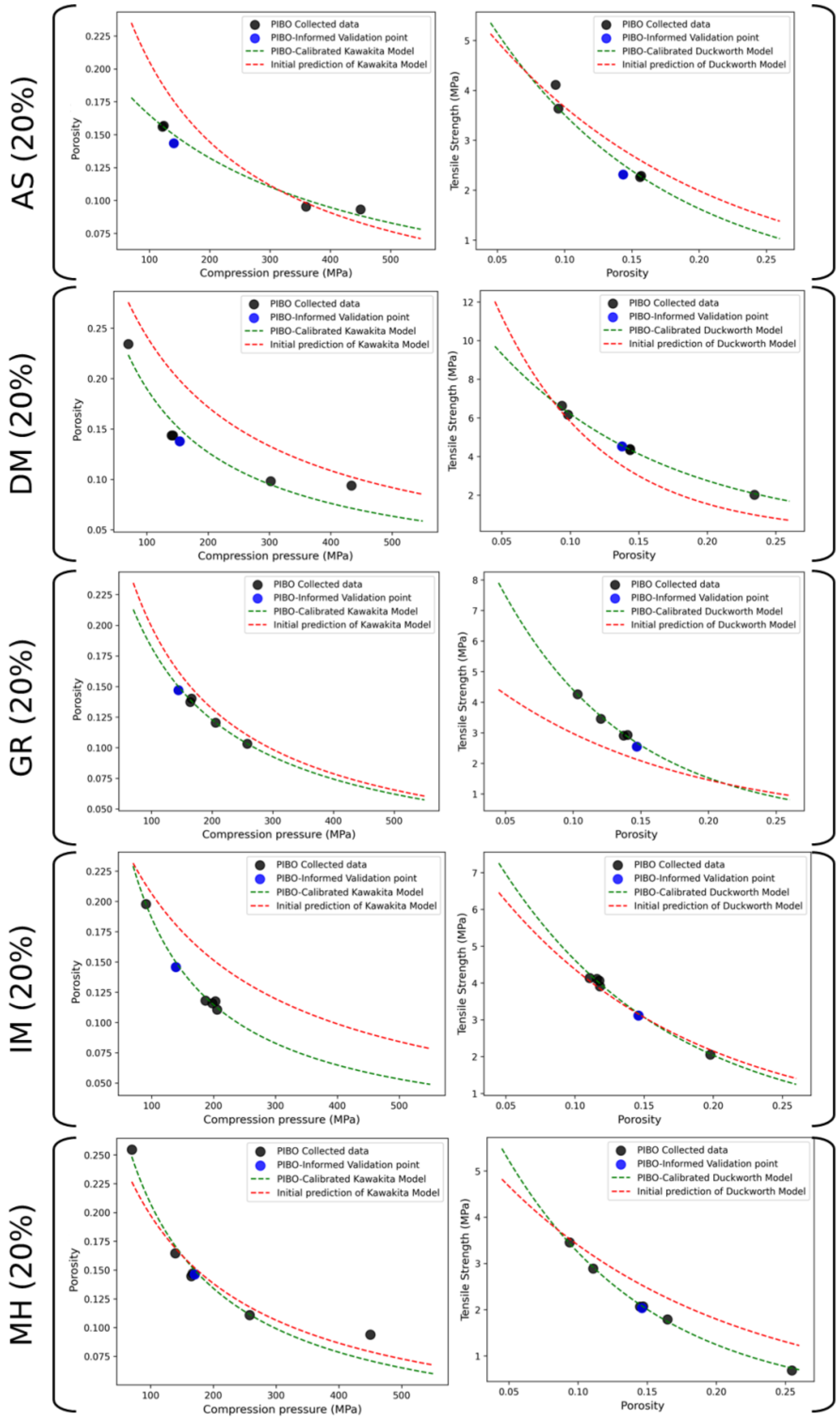

Supplementary Figure 38 (Cont'd): Initial and calibrated prediction of compressibility and compactability profiles before (using the system of models) and after the calibration with PIBO. Source data are provided as a Source Data file.

Supplementary Figure 39 shows the reliability and generalizability of the PIBO across a diverse set of formulations using tableting data factory. Supplementary Figure 39a demonstrates that the system is able to consistently produce tablets whose final weights fall within the predefined target range, regardless of the active pharmaceutical ingredient (API) or concentration used in

the formulation in these use cases. This highlights the robustness of the dosing and compaction process to meet the specified weight constraints across the nine use cases. Supplementary Figure 39b and c validate the ability of the optimisation workflow to achieve the desired porosity and tensile strength. Despite substantial variation in material properties across APIs and excipient ratios, the system is able to converge on compaction pressures and process conditions that yield tablets with 15% porosity and more than 2 MPa tensile strength.

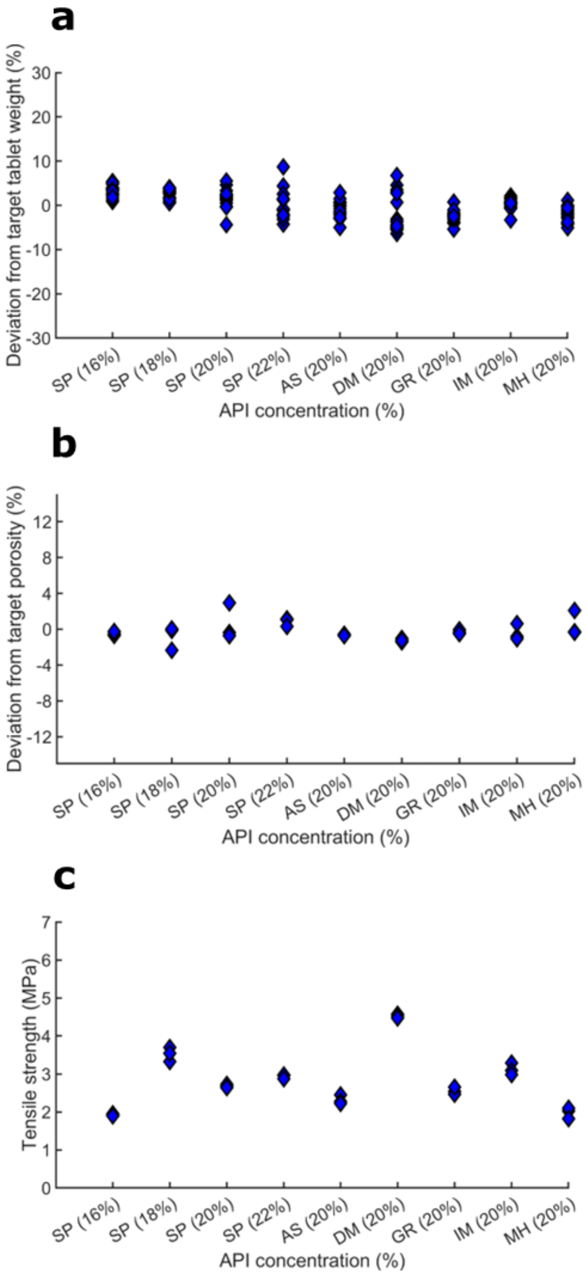

Supplementary Figure 39: Validation of optimised target **a** weight, **b** porosity and **c** tensile strength of tablets with different APIs and concentration. Source data are provided in the Source Data file.

4.3.3 Multi-output Bayesian optimisation (MOBO) for rapid scale-up assessment

During scale-up assessment of tableting data factory, process parameters can behave differently due to increased speeds and forces, requiring careful consideration of intercorrelation between multiple process parameters [2]. A scale-up, multi-output Bayesian framework was designed to minimise elastic recovery while achieving target porosity and tensile strength by optimising key process parameters such as main compression pressure, precompression pressure, and dwell time. Minimising elastic recovery, although is not considered in the digital formulator and hybrid system of models due to the absence of required training and validation data, is critical in process optimisation and refinement, as it is closely associated with tablet defects such as lamination, capping, and air entrapment, particularly when scaling up from compaction simulators to rotary tablet presses [17, 18].

The proposed MOBO was tested on two formulations, SP (20%) and AS (20%), which were identified using the digital formulator. The MOBO was set for exploration, allowing for the collection of sufficient data to effectively train the GP models across a large parameter space (Supplementary Figure 40). Three individually trained GP models can then be used to predict elastic recovery, tensile strength, and porosity across varying precompression, main compression pressures, and dwell times. This predictive knowledge space is refined to identify a manufacturability region, where tensile strength exceeds 2 MPa and porosity remains above 0.15, as shown in Figure 41. Within this region, the optimal main compression and precompression pressures can be determined to minimise elastic recovery while meeting porosity and tensile strength constraints.

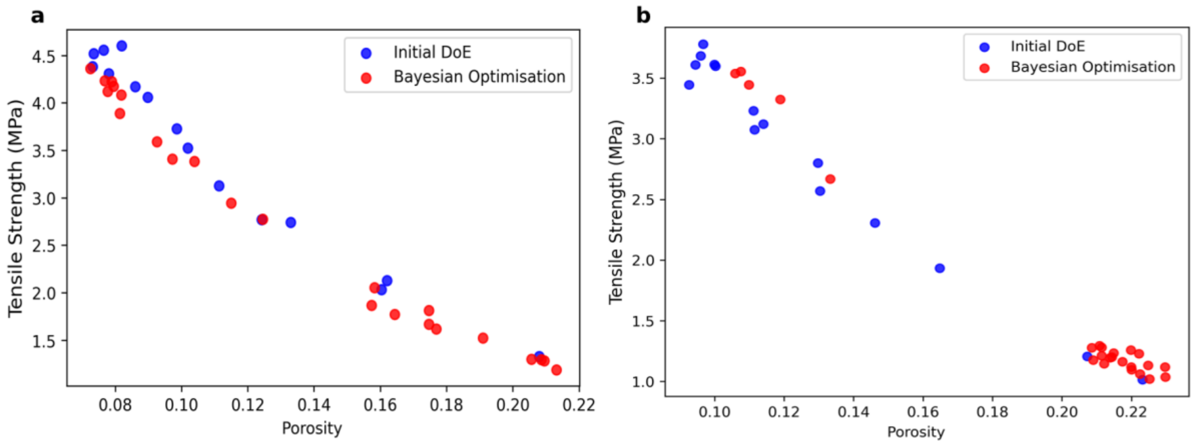

Supplementary Figure 40: Representation of compactability profile of **a** SP (20 wt.%) and **b** AS (20 wt.%) formulations using the collected data points during MOBO. Source data are provided as a Source Data file.

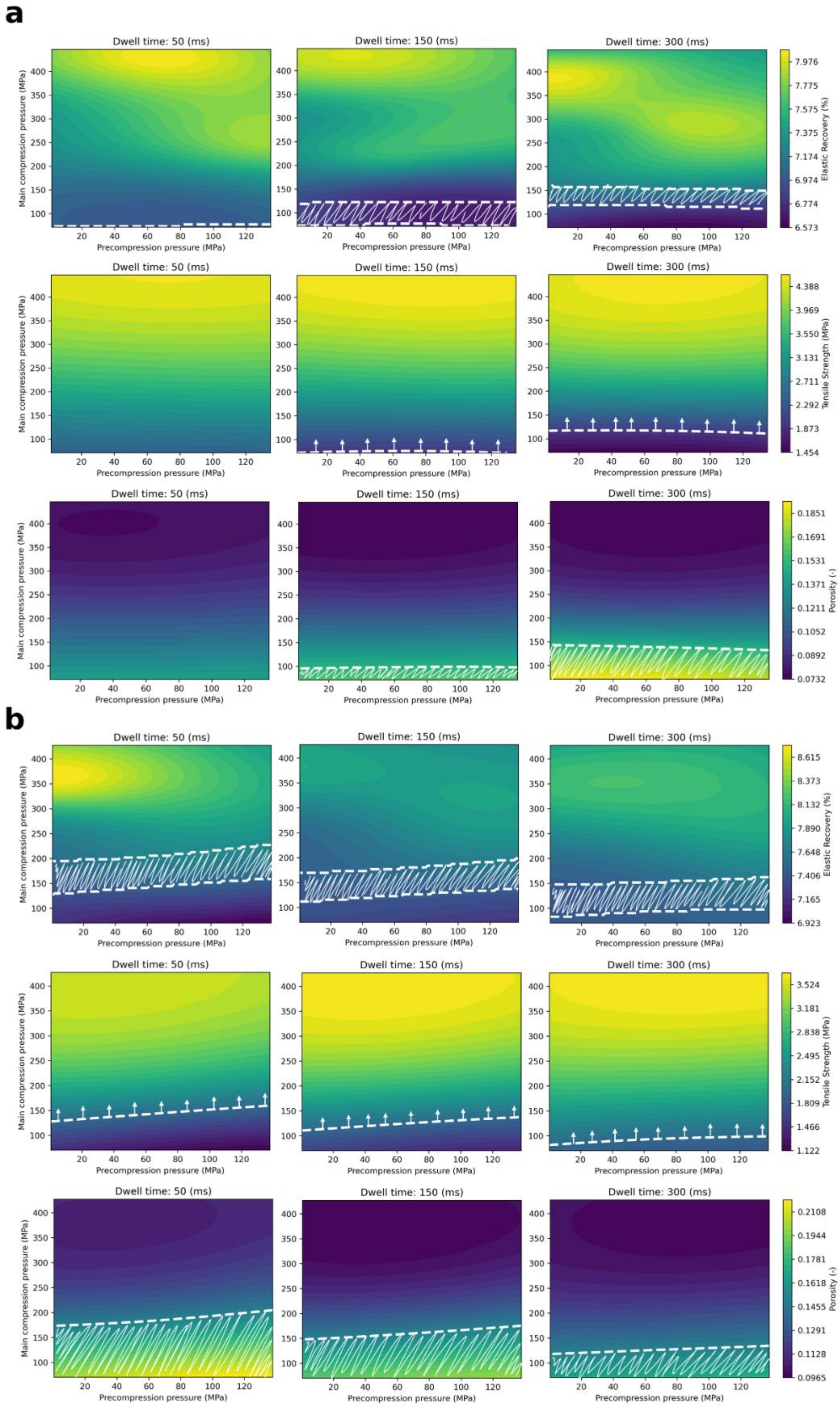

Figure 41: Heatmaps generated by self-driving tableting data factory using MOBO approach. The heatmaps show elastic recovery (top row), tensile strength (middle row), and porosity

(bottom row) based on the variation of precompression and main compression pressure at three different dwell times (50, 150, 300 ms) for **a** SP (20 wt.%) and **b** AS (20 wt.%) case study. For each case study, in the bottom row, the regions filled within the dashed lines correspond to the porosity ≥ 0.15. In the middle row, the arrows in the middle-row figures indicate regions (i.e., above the dashed line) where specific criteria are met, such as the area at the top satisfying tensile strength ≥ 2 MPa. In the top row, the region filled within the dashed lines corresponds to the area that both porosity and tensile strength constrains are satisfied. Source data are provided as a Source Data file.

4.3.4 Disintegration testing

To evaluate whether the tablets produced using the PIBO approach also meet performance criteria, we conducted disintegration testing on tablets manufactured from the nine use cases blends. For each blend, PIBO was used to identify the compaction pressure that would yield tablets with ≈ 15% porosity and tensile strength more than 2 MPa. The selected pressures for these nine use cases are summarised in Supplementary Table 9 and correspond to the validation points highlighted in blue in Supplementary Figure 37.

Using these PIBO-recommended pressures, six tablets were produced for each formulation, resulting in a total of fifty-four tablets prepared for disintegration analysis. This experimental design allows us to assess not only whether PIBO can achieve the targeted physical attributes, but also whether tablets meeting those targets exhibit acceptable disintegration performance, a key quality attribute for oral solid dosage forms.

Supplementary Figure 42 presents the disintegration time measurements for the tablets across all nine blends. The results demonstrate how differences in API physicochemical properties and excipient interactions influence disintegration behaviour, even when porosity and tensile strength are controlled to consistent target values. These findings provide an important validation step, confirming that the PIBO-derived compaction pressures produce tablets with both the intended mechanical properties and functionally relevant disintegration times.

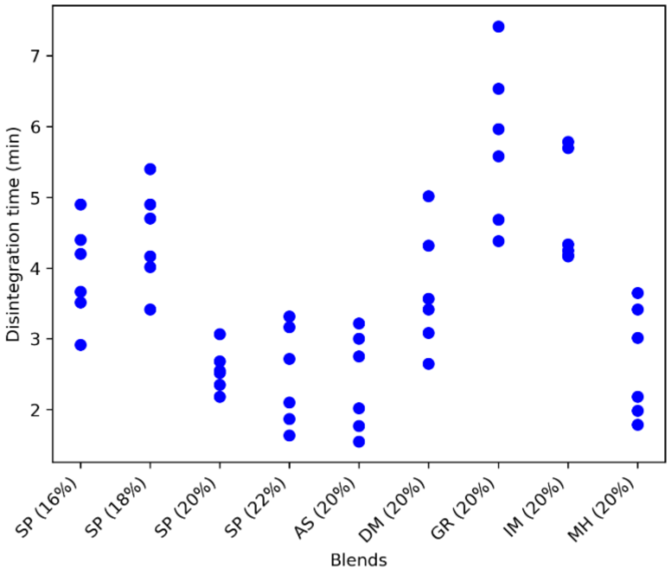

Supplementary Figure 42: Disintegration testing on tablets with 15% porosity and > 2 MPa tensile strength for the nine use cases listed in Supplementary Table 9. Six tablets were manufactured from each of the nine formulation blends, using the compaction pressures recommended by the PIBO approach. These validation pressure points are highlighted in blue in Supplementary Figure 37-38. Source data are provided in the Source Data file.

5 Tablet manufacturing

The tableting data factory can be operated in a manufacturing mode to deliver a consistent batch of tablets that meet the quality standards. Considering the current speed of the system, it can deliver 1,440 tablets within 24 h with real-time monitoring of each tablet produced.

5.1 Extended reality enabled process monitoring

The system generates multi-dimensional data for every single tablet necessitating novel approaches to support human-driven, data-centric quality monitoring and decision-making. This was achieved by integrating EXTENDED REALITY technology, specifically augmented reality (AR) and mixed reality (MR) into the tableting data factory to connect physical assets, data and the researcher in an intuitive, accessible and effective manner. AR is a technology that overlays digital content onto the real world, enhancing the user's perception of their environment. MR is the blend of physical and digital worlds where virtual objects interact with real-world elements in real time. However, MR is an umbrella term encompassing AR and MR covering all immersive technologies that merge digital and physical experiences. Both AR and MR platforms visualise a dashboard with the key quality parameters, specifically tablet weight, porosity, and tensile strength in real-time, whilst also streaming real-time data of individual instruments as shown in Supplementary Figure 43.

AR is utilised in the lab to overlay real-time data directly onto individual instruments. This allows researchers and operators to access crucial information, such as performance metrics, operational statuses, and diagnostic data, without the need to refer to external displays.

The MR version is specifically designed to allow users to visualise experimental data in real-time outside of the laboratory environment. The MR hologram of the tableting data factory provides an immersive, 3D representation of the whole system in operation. This holographic visualisation allows users to virtually observe the production process, inspect the parameters from individual instruments, and troubleshoot potential issues from a remote location. The integration of MR technology thus extends the accessibility and control over the system, significantly improving collaboration, training methods and oversight in a distributed work environment and provides further opportunities for user training. Future work aims to integrate real-time quality control (QC) using extended reality, leveraging the existing holographic infrastructure.

The AR and MR applications in the tableting data factory are demonstrated in the Supplementary Videos 2 and 3, respectively. These examples demonstrate continuous production of 100 tablets. The immersive overlaid diagrams in both AR and MR are designed to highlight the acceptable (green) and unacceptable (red) data points. The acceptable range for each of these parameters is ±5% of defined target. The progress of the overall manufacturing stage is shown by visualising the total tablets produced, stored and analysed. Data of these 100 tablets is also shown in Supplementary Figure 44.

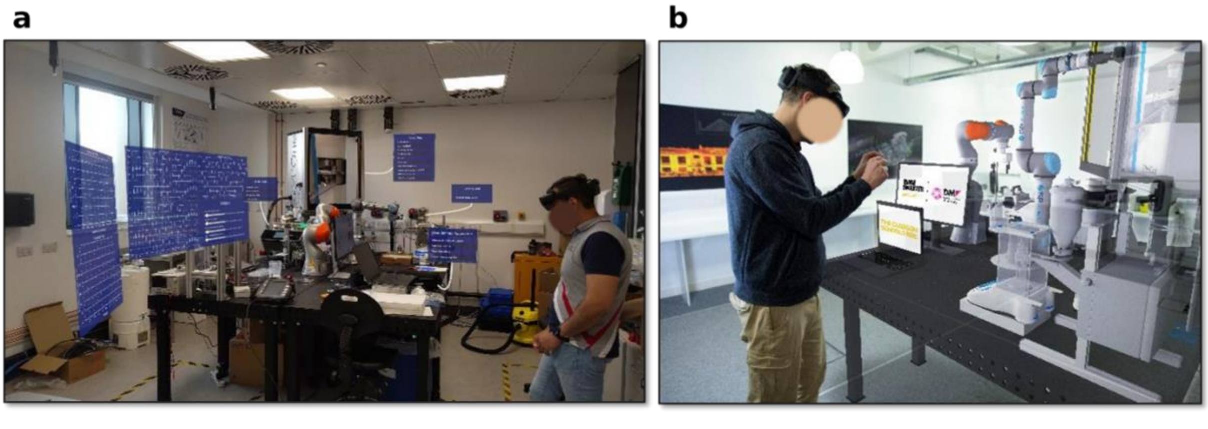

Supplementary Figure 43: **a** AR – real-time experimental data overlaid on corresponding equipment during experiment. **b** MR – Real-time display of holographic equipment for remote laboratory access.

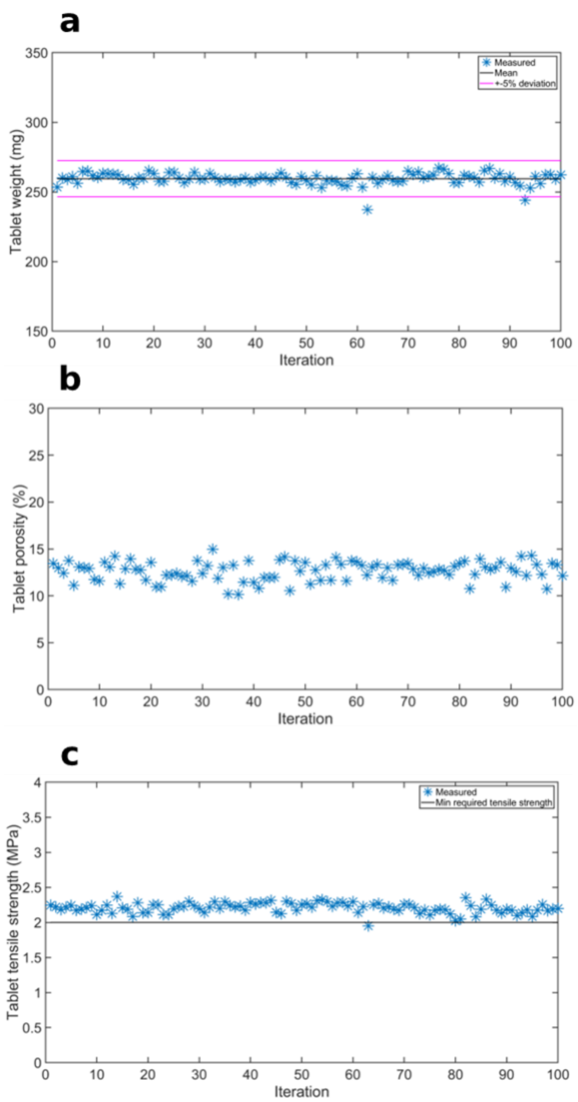

Supplementary Figure 44: Assessment of variability in tablet **a** weight, **b** porosity and **c** tensile strength over time. This assessment is performed on 100 tablets that are produced continuously at a fixed compression pressure using SP (16 wt.%) formulation. Source data are provided in the Source Data file.

5.2 Implementation of extended reality

An immersive digital twin of the tableting data factory was implemented using a real-time development platform (Unity) targeting Microsoft's HoloLens 2 mixed reality devices. 3D models of the tableting data factory equipment were constructed with Autodesk's 3D Studio Max using 3D CAD files, brochures, photos and measurements taken on site. The 3D lab models were then imported into the Unity environment for uploading to the HoloLens devices. To gather the data from the tableting data factory, a REST API (allowing systems to exchange data over the internet) was implemented to take advantage of the integration capabilities of LabVIEW. The HoloLens can access the experimental data in real-time as long as it is connected online. This enables the user to view real-time experimental data using either the

AR digital twin at the lab (data overlaid onto real lab equipment), or the MR digital twin (laboratory hologram) while working remotely.
The AR version of the digital twin aligns all the overlaid data onto the associated equipment within the tableting data factory. This is achieved using a single QR code that the HoloLens detects and uses as a reference to calibrate the entire scene.
The MR version of the digital twin can be used in any location. It uses surface (floor) detection enabling the user to place a scalable tableting data factory hologram into any suitable location. The digital twin enables users to receive real-time updates to the lab's holographic representation and data overlays, even when they are located remotely from the physical lab.

6 Comparison to the state of the art

This study was compared to two alternative state-of-the art methods from the literature that optimise the calibration of compressibility and compactability models. These studies also focused on reducing the experimentation required to predict these behaviours for a given excipient-API (fixed excipient choice for binder/filler 1 and 2, lubricant and disintegrant) formulation space. A comparison of the API mass required for the calibration of these state-of-the-art modelling methods was conducted (Supplementary Table 15). Corrigan, Li [20] employed a linear mixture model with the addition of binary interaction terms to predict the mixture behaviours based on the formulations composition. Jolliffe, Ojo [21] proposed a combined modelling and experimental approach to determine theoretical pure component tuning parameters to reduce the experimental burden for the parameterisation of mixture models. Both of these literature studies require multiple formulated tablets with API to calibrate their modelling method to gain a better understanding of a design space. For consistency in comparing these methodologies to this study, the tablet masses have been set to 250 mg as per this study. At time of writing, Corrigan's method was found to be the best performing approach in the literature to reduce experimentation and thus has been set as the benchmark for comparison between methods.
It was found that the platform workflow with the digital formulator and the tableting data factory was capable of achieving a satisfactory prediction of compressibility and compactability behaviours with a 65% reduction in API mass required. There is a discrepancy between these compared methodologies in the quantity of tablets manufactured at each compression point which will impact the mass of API required for model population. This has been kept as cited on the assumption that this may also have an impact on model accuracy. Corrigan, Li [20] performed 10 pre-determined pressure points with three tablets at each pressure point. Importantly, one of the key differences between our method and Corrigan, et al. is that, instead of producing and testing three tablets at several pre-determined pressure points, this study uses a Bayesian optimisation–guided process to identify pressure points adaptively leading to a reduced number of pressure points and tablets. This enables the calibration of a reliable model for the tensile strength–porosity relationship using only 4-5 pressure points with three tablets per pressure point without compromising model accuracy.
This comparison was conducted on the basis of demonstrating the reduction of API mass required to achieve a formulation which can be manufactured successfully. It is evident that this study outperforms the state-of-the-art methodologies in this instance, however if the goal of the study is to gain understanding of the compressibility and compactability behaviours of a whole ternary formulation space (1 API and 2 excipients) across the selected excipient-API combination, Corrigan, Li [20] method was shown to be also very valuable.

Supplementary Table 15: Comparison of API consumption between this study and two state of the art approaches. Supplementary Table 16 provides details on the material use for the raw material characterisation.

	This study (digital formulator only)	This study (digital formulator and tableting data factory)	Corrigan, Li [20]	Jolliffe, Ojo [21]
API mass required for raw material characterisation (g)	1.90	1.90	-	-
Tablet mass (mg)	-	250	250	250
API concentration required for calibration (wt.%)	-	20	20, 40, 40	10, 20, 40
Number of compression points considered	-	5	10	8
Number of tablets per compression point	-	3	3	10
Total number of tablets made	-	15	30	80
Model refinement and calibration (g)	-	0.75	7.5	14
Total mass of API consumed (g)	1.90	2.65	7.5	14
Total percentage of API consumed compared to Corrigan, Li [20] (%)	-75	-65	0	87

The total mass of API, m_T, required to calibrate and refine the modelling approaches was estimated as follows:

$$m_T = Nm\sum_{i=1}^{k} c_i \qquad \text{Supp. Equation (16)}$$

This accounts for the number of tablets, N, manufactured at tablet mass, m, of API mass concentration, c_i, where the subscript i accounts for the cases where multiple formulations, k, were required. Once m_T was estimated for each study, $m_{T,j}$ they were compared to the mass used by Corrigan, Li [20], $m_{t,\text{Corrigan}}$,to achieve a percentage difference, Δm_T:

$$\Delta m_T = 100\frac{m_{T,j} - m_{T,\text{Corrigan}}}{m_{T,\text{Corrigan}}} \qquad \text{Supp. Equation (17)}$$

API mass required for the material characterization for the digital formulator is given in Table

Supplementary Table 16: Summary of amount of material used for each required measurement. These measurements are essential inputs to the digital formulator. These values vary slightly between materials; a rounded average is reported here. True density is measured using gas pycnometry with a 0.25 cm^3 sample cell. Bulk density is measured using a measuring cylinder with a volume of 0.543 cm^3. Particle size/shape is measured using a static imaging method which was a Morphologi G4 (Malvern Panalytical, Malvern, UK) in this study.

Characteristic	Amount used per repetition (g)	No of Repeats	Total amount used (g)
True density	0.3	3	0.9
Bulk density	0.3	3	0.9
Particle size/shape	0.1	1	0.1
		**Total (g)**	**1.9**

Supplementary references

1 Salehian, M. et al. A hybrid system of mixture models for the prediction of particle size and shape, density, and flowability of pharmaceutical powder blends. *Int. J. Pharm. X* **8**, 100298 (2024).

2 Vreeman, G. & Sun, C. C. A strategy to optimize precompression pressure for tablet manufacturing based on in-die elastic recovery. *Int. J. Pharm.* **654**, 123981 (2024).

3 Meynard, J., Amado-Becker, F., Tchoreloff, P. & Mazel, V. On the complexity of predicting tablet capping. *Int. J. Pharm.* **623**, 121949 (2022).

4 Mazel, V. & Tchoreloff, P. Lamination of pharmaceutical tablets: classification and influence of process parameters. *J. Pharm. Sci.* **111**, 1480–1485 (2022).

5 Kawakita, K. & Tsutsumi, Y. An empirical equation of state for powder compression. *Jpn. J. Appl. Phys.* **4**, 56 (1965).

6 Duckworth, W. Discussion of Ryshkewitch paper by Winston Duckworth. *J. Am. Ceram. Soc.* **36**, 68–69 (1953).

7 LeCun, Y., Bengio, Y. & Hinton, G. Deep learning. *Nature* **521**, 436–444 (2015).

8 Breiman, L. Random forests. *Mach. Learn.* **45**, 5–32 (2001).

9 Drucker, H., Burges, C. J., Kaufman, L., Smola, A. & Vapnik, V. Support vector regression machines. in *Adv. Neural Inf. Process. Syst.* **9**, 155–161 (MIT Press, 1996).

10 Sykes, R. A. et al. What has scripting ever done for us? The CSD Python application programming interface (API). *J. Appl. Crystallogr.* **57**, 1235–1250 (2024).

11 Moldovan, A. A. & Maloney, A. G. Surface Analysis—From Crystal Structures to Particle Properties. *Cryst. Growth Des.* **24**, 4160–4169 (2024).

12 Shahriari, B., Swersky, K., Wang, Z., Adams, R. P. & De Freitas, N. Taking the human out of the loop: A review of Bayesian optimization. *Proc. IEEE* **104**, 148–175 (2016).

13 Williams, C. K. & Rasmussen, C. E. *Gaussian Processes for Machine Learning* (MIT Press, 2006).

14 Snoek, J., Larochelle, H. & Adams, R. P. Practical Bayesian optimization of machine learning algorithms. in *Adv. Neural Inf. Process. Syst.* **25**, 2951–2959 (Curran Associates, 2012).

15 Brochu, E., Cora, V. M. & De Freitas, N. A tutorial on Bayesian optimization of expensive cost functions, with application to active user modeling and hierarchical reinforcement learning. *arXiv preprint arXiv:1012.2599* (2010).
16 Jones, D. R., Schonlau, M. & Welch, W. J. Efficient global optimization of expensive black-box functions. *J. Glob. Optim.* **13**, 455–492 (1998).
17 Pitt, K. & Sinka, C. Tabletting. in *Handbook of Powder Technology* Vol. 11 (eds Salman, A. D., Ghadiri, M. & Hounslow, M. J.) 495–539 (Elsevier, 2007).
18 Iurian, S., Casian, T., Porfire, A. & Tomuţă, I. Tablet compression and consolidation: theory and applications. in *Physico-Chemical Aspects of Dosage Forms and Biopharmaceutics* (eds Wiedmann, T. S. & Zhang, Y.) 285–326 (Academic Press, 2024).
19 Loh, W.-L. On Latin hypercube sampling. *Ann. Stat.* **24**, 2058–2080 (1996).
20 Corrigan, J. et al. An interaction-based mixing model for predicting porosity and tensile strength of directly compressed ternary blends of pharmaceutical powders. *Int. J. Pharm.* **664**, 124587 (2024).
21 Jolliffe, H. G. et al. Linked experimental and modelling approaches for tablet property predictions. *Int. J. Pharm.* **626**, 122116 (2022).
