# Supplementary material for: Accelerated drug development using a digital formulator and a self-driving tableting data factory
Source: Nat Commun. 2026 Apr 1;17:4739. doi: 10.1038/s41467-026-71204-6 (PMC13216267; doi:10.1038/s41467-026-71204-6)
Supplement: Supplementary file 2 — Description of Additional Supplementary Files [file 41467_2026_71204_MOESM2_ESM.pdf]

File name: Supplementary Movie 1
Description: Operation of the self-driving tableting data factory. This video demonstrates the full operation of the self-driving tableting data factory, including automated powder dosing, in-line weight measurement, near-infrared analysis, powder transport, tablet compaction using a tablet press, and subsequent tablet testing.

File name: Supplementary Movie 2
Description: Augmented reality demonstration of the tableting data factory in the laboratory. This video, recorded from a Microsoft HoloLens perspective, demonstrates the augmented reality interface of the tableting data factory, where real-time operational data from individual instruments are overlaid onto their physical counterparts. The display includes tablet quality parameters such as weight, tensile strength, and porosity across multiple iterations, as well as tablet compressibility and compactability profiles.

File name: Supplementary Movie 3
Description: Mixed reality demonstration of the tableting data factory outside the laboratory. This video, recorded from a Microsoft HoloLens perspective, demonstrates the mixed reality interface of the tableting data factory in a remote setting outside the laboratory. Operational data from individual instruments are overlaid onto their corresponding 3D virtual models. The display includes tablet quality parameters such as weight, tensile strength, and porosity across multiple iterations, as well as tablet compressibility and compactability profiles.
