# Supplementary material for: Accelerated drug development using a digital formulator and a self-driving tableting data factory
Source: Nat Commun. 2026 Apr 1;17:4739. doi: 10.1038/s41467-026-71204-6 (PMC13216267; doi:10.1038/s41467-026-71204-6)
Supplement: Supplementary file 6 — Reporting Summary [file 41467_2026_71204_MOESM6_ESM.pdf]

nature portfolio

Corresponding author(s): Dr Daniel Markl

Last updated by author(s): Feb 13, 2026

Reporting Summary

Nature Portfolio wishes to improve the reproducibility of the work that we publish. This form provides structure for consistency and transparency in reporting. For further information on Nature Portfolio policies, see our Editorial Policies and the Editorial Policy Checklist.

Statistics

For all statistical analyses, confirm that the following items are present in the figure legend, table legend, main text, or Methods section.

n/a	Confirmed	
☐	☒	The exact sample size (*n*) for each experimental group/condition, given as a discrete number and unit of measurement
☐	☒	A statement on whether measurements were taken from distinct samples or whether the same sample was measured repeatedly
☒	☐	The statistical test(s) used AND whether they are one- or two-sided<br>*Only common tests should be described solely by name; describe more complex techniques in the Methods section.*
☒	☐	A description of all covariates tested
☒	☐	A description of any assumptions or corrections, such as tests of normality and adjustment for multiple comparisons
☐	☒	A full description of the statistical parameters including central tendency (e.g. means) or other basic estimates (e.g. regression coefficient) AND variation (e.g. standard deviation) or associated estimates of uncertainty (e.g. confidence intervals)
☒	☐	For null hypothesis testing, the test statistic (e.g. *F*, *t*, *r*) with confidence intervals, effect sizes, degrees of freedom and *P* value noted<br>*Give P values as exact values whenever suitable.*
☒	☐	For Bayesian analysis, information on the choice of priors and Markov chain Monte Carlo settings
☒	☐	For hierarchical and complex designs, identification of the appropriate level for tests and full reporting of outcomes
☒	☐	Estimates of effect sizes (e.g. Cohen's *d*, Pearson's *r*), indicating how they were calculated

Our web collection on statistics for biologists contains articles on many of the points above.

Software and code

Policy information about availability of computer code

Data collection	This study utilizes custom algorithms and codes that are central to the research, all source data and codes are deposited in the repository accessible to editor and reviewers.<br><br>All the data from DataFactory is acquired using LabVIEW. Instruments' digital integration is explained in the manuscript and supporting information. LabVIEW codes are also deposited in the repository.<br><br>Link: https://gitlab.cis.strath.ac.uk/fmb22104/cmac-self-driving-tabletting-datafactory
Data analysis	This study utilizes custom algorithms and codes that are central to the research, all source data and codes are deposited in the repository accessible to editor and reviewers. Link: https://gitlab.cis.strath.ac.uk/fmb22104/cmac-self-driving-tabletting-datafactory

For manuscripts utilizing custom algorithms or software that are central to the research but not yet described in published literature, software must be made available to editors and reviewers. We strongly encourage code deposition in a community repository (e.g. GitHub). See the Nature Portfolio guidelines for submitting code & software for further information.

Data

Policy information about availability of data

All manuscripts must include a data availability statement. This statement should provide the following information, where applicable:

- Accession codes, unique identifiers, or web links for publicly available datasets
- A description of any restrictions on data availability
- For clinical datasets or third party data, please ensure that the statement adheres to our policy

Our data and code availability statement provides a link to our source data and code. The source data provides data to reproduce all display items in the submission. The data and codes with comprehensive instructions are provided in the repository: https://gitlab.cis.strath.ac.uk/fmb22104/cmac-self-driving-tabletting-datafactory

Research involving human participants, their data, or biological material

Policy information about studies with human participants or human data. See also policy information about sex, gender (identity/presentation), and sexual orientation and race, ethnicity and racism.

Reporting on sex and gender	N/A
Reporting on race, ethnicity, or other socially relevant groupings	N/A
Population characteristics	N/A
Recruitment	N/A
Ethics oversight	N/A

Note that full information on the approval of the study protocol must also be provided in the manuscript.

Field-specific reporting

Please select the one below that is the best fit for your research. If you are not sure, read the appropriate sections before making your selection.

[x] Life sciences [ ] Behavioural & social sciences [ ] Ecological, evolutionary & environmental sciences

For a reference copy of the document with all sections, see nature.com/documents/nr-reporting-summary-flat.pdf

Life sciences study design

All studies must disclose on these points even when the disclosure is negative.

Sample size	No formal sample size calculation was performed for this study. Sample sizes were determined based on resource availability, practical experimental design considerations, and established practices in pharmaceutical development research.
Data exclusions	No data exclusions were implemented in this study. All provided data points were included in the analysis and modeling procedures.
Replication	All experiments were performed at least in triplicate. For each formulation case, powder dosing, compaction and testing steps were repeated three times, with the mean of the three measurements used for model inputs and optimisation. Disintegration tests used six tablets per blend (two tablets from each of three replicate runs). NIR-based blend-tablet uniformity assessments acquired five subsamples per blend, each processed as three replicate scans. This triplicate replication at every key stage ensured robust, repeatable results.
Randomization	Randomization is not applicable to this study. This research employed a computational and process optimisation framework rather than clinical or controlled experimental design requiring subject randomization. The study focused on material characterization, predictive modeling, and automated manufacturing processes where experimental conditions were systematically varied through optimization approaches rather than random assignment of subjects or treatments.
Blinding	Blinding is not relevant to this study. This work employed automated instrumentation and objective analytical measurements (e.g., tablet weight, porosity, tensile strength) rather than subjective assessments that could be influenced by investigator bias.

Reporting for specific materials, systems and methods

We require information from authors about some types of materials, experimental systems and methods used in many studies. Here, indicate whether each material, system or method listed is relevant to your study. If you are not sure if a list item applies to your research, read the appropriate section before selecting a response.

Materials & experimental systems

n/a	Involved in the study
☒	☐ Antibodies
☒	☐ Eukaryotic cell lines
☒	☐ Palaeontology and archaeology
☒	☐ Animals and other organisms
☒	☐ Clinical data
☒	☐ Dual use research of concern
☒	☐ Plants

Methods

n/a	Involved in the study
☒	☐ ChIP-seq
☒	☐ Flow cytometry
☒	☐ MRI-based neuroimaging

Plants

Seed stocks	N/A
Novel plant genotypes	N/A
Authentication	N/A
